# Interspecific and intraspecific phenotypic diversity for drought adaptation in bioenergy *Arundo* species

**DOI:** 10.1111/gcbb.12810

**Published:** 2021-02-16

**Authors:** Michele Faralli, Kevin Williams, Fiona Corke, Mingai Li, John H. Doonan, Claudio Varotto

**Affiliations:** ^1^ Department of Biodiversity and Molecular Ecology, Research and Innovation Centre Fondazione Edmund Mach San Michele all'Adige (TN) Italy; ^2^ National Plant Phenomics Centre (NPPC) IBERS Aberystwyth University Wales UK

**Keywords:** *Arundo*, gas exchange, giant reed, high‐throughput phenotyping, vapour pressure deficit, water stress

## Abstract

Biomass crops are commonly grown in low‐grade land and selection of drought‐tolerant accessions is of major importance to sustain productivity. In this work, we assess phenotypic variation under different environmental scenarios in a series of accessions of *Arundo donax*, and contrast it with two closely related species, *Arundo donaciformis* and *Arundo plinii*. Gas‐exchange and stomatal anatomy analysis showed an elevated photosynthetic capacity in *A*. *plinii* compared to *A. donax* and *A. donaciformis* with a significant intraspecific variation in *A. donax*. The three species showed significantly contrasting behaviour of transpiration under developing water stress and increasing vapour pressure deficit (VPD), with *A. donax* being the most conservative while *A*. *plinii* showed an elevated degree of insensitivity to environmental cues. Under optimal conditions, *A. donax* had the highest estimated leaf area (projected leaf area) and plant dry weight although a significant reduction under water stress was observed for *A. donax* and *A. donaciformis* accessions while no differences were recorded for *A*. *plinii* between optimal growing conditions (well‐watered [WW]) and reduced soil water availability (water‐stressed [WS]). *A. donax* displayed a markedly conservative water use behaviour but elevated sensitivity of biomass accumulation under stress conditions. By contrast, in *A*. *plinii*, biomass and transpiration were largely insensitive to WS and increasing VPD, though biomass dry weight under optimal conditions was significantly lower than *A. donax*. We provide evidence of interspecific phenotypic variation within the *Arundo* genus while the intraspecific phenotypic plasticity may be exploited for further selection of superior clones under disadvantageous environmental conditions. The extensive trade‐off between water use and biomass accumulation present in the three species under stress conditions provides a series of novel traits to be exploited in the selection of superior clones adapted to different environmental scenarios. Non‐destructive approaches are provided to screen large populations for water‐stress‐tolerant *A. donax* clones.

## Graphical abstract

We compared by high‐throughput phenotyping natural populations of three species of the *Arundo* genus, the giant reed *Arundo donax* and two of its wild relatives, *Arundo donaciformis* and *Arundo plinii*, for drought tolerance. A clearly water‐conservative behavior was observed in *A. donax* and *A. donaciformis*. Conversely, *A*. *plinii* displayed an overall non‐conservative response to developing water stress. The intraspecific variation we uncovered in *A. donax* suggests the possibility of selecting natural ecotypes for agronomic use with superior combinations of preferable traits for different environments. In addition, our work stresses the importance of non‐destructive phenotyping approaches for plant‐environment interaction studies.

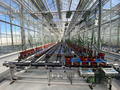

## 1 INTRODUCTION

Soil water deficit is one of the most limiting environmental factors for plant growth and reproduction (Lesk et al., 2016). It is now well established that the exacerbation of adverse conditions resulting from a changing climate, in particular water stress, will reduce the area of available land for agriculture by 11%–17% within Europe (Stavridou et al., 2019). A promising option to reduce competition for high‐grade land is growing bioenergy crops on land unsuitable for food crops (Popp et al., 2014; Somerville et al., 2010). This alternative, however, is based on the assumption that expensive management practices (e.g. irrigation) will not be established in low‐grade areas and therefore development of bioenergy crops that are more productive with less water requires advances in understanding the physiological basis underlying stress tolerance in bioenergy crops of interest (Cosentino et al., 2014; Stavridou et al., 2019).

*Arundo donax* is the most promising species for bioenergy purposes in the Mediterranean basin (Cosentino et al., 2014; Nazli et al., 2018; Zegada‐Lizarazu et al., 2020) owing to its high yield potential (Cosentino et al., 2014; Zegada‐Lizarazu et al., 2020) and sustained salt tolerance (Nacley & Kim, 2015). Recent transcriptomic work laid a solid foundation regarding stress physiology and high productivity in *A. donax* (e.g. Barrero et al., 2015; Evangelistella et al., 2017; Fu et al., 2016; Sablok et al., 2014; Sicilia et al., 2019). However, *A. donax* is very sensitive to limited water conditions (Haworth, Cosentino, et al., 2017; Pompeiano et al., 2017; Zegada‐Lizarazu et al., 2020) with severe reduction both in physiological performance (Haworth, Cosentino, et al., 2017; Mann et al., 2013) and in biomass production (up to 20%; Zegada‐Lizarazu et al., 2020) under water stress. Indeed, the elevated water requirement limits its potential cultivation in marginal agricultural areas and improving water stress tolerance in *A. donax* is an important goal for the future.

Breeding germplasm adapted to water deficit that can maintain or boost productivity in drought‐prone areas relies on the use of favourable traits within breeding programmes. Conventional breeding schemes have been successful in many food (Lee & Tollenaar, 2007; William et al., 2007) and biomass (Clifton‐Brown et al., 2019) crops. However, *A. donax* is generally thought to be sterile (Mariani et al., 2010) and thus is characterized by limited genetic diversity (Hardion et al., 2012; Hardion et al., 2014). Since conventional crop improvement cannot be implemented, mutagenesis (e.g. Valli et al., 2017) and ecotype selection remain the main sources of phenotypic diversity. Significant epigenetic diversity exists within Italian *A. donax* clones (Guarino et al., 2019), suggesting that the capacity of *A. donax* to thrive in a vast array of environmental conditions may be partially explained by different DNA methylation levels. Other species belonging to the same genus, for example, *Arundo donaciformis* and *Arundo plinii*, produce seed and display an elevated degree of drought tolerance (Danin, 2004). Despite *A. donaciformis* and *A*. *plinii* being phylogenetically sister species (Jike et al., 2020), *A. donaciformis* underwent the same number of demiploidization events as *A. donax* (Jike et al., 2020) and attains a larger size than *A*. *plinii* (Hardion et al., 2014). Thus, a comparative assessment of *A. donax*, *A. donaciformis* and *A*. *plinii* could provide additional insights into the relationship between productivity, drought tolerance and polyploidization level. *A. donaciformis* and *A*. *plinii* would be relatively ineffective sources of biofuel due to their low biomass production although, by generally growing in very marginal areas, they may provide useful information on preferable traits to optimize and further develop superior *A. donax* ecotypes for environmental stress tolerance.

Under rain‐fed or ‘Mediterranean‐type’ environments where recurrent periods of water stress occur during the growing seasons, selection should tend to favour traits that promote water saving until the next rain episode (Sadok & Tamang, 2019; Schoppach & Sadok, 2012). Since high stomatal resistance and pronounced intrinsic water‐use efficiency can lead to significant reduction in carbon assimilation following a restricted capacity for gas exchange (Blum, 2005; Sinclair, 2018), superior and timely responses to environmental stimuli should be key characters for selecting highly water‐stress‐tolerant crops. In wheat and other cereals, as well as in other fruit crops (e.g. grapevine, Tombesi et al., 2015), two key physiological traits have been identified to select germplasm with superior water stress tolerance: (i) a limited transpiration rate (TR) to a constant, maximum value under high levels of air vapour pressure deficit (VPD) when the soil moisture is high and (ii) an early TR decrease at a high threshold of transpirable soil water (Schoppach & Sadok, 2012; Sinclair, 2018; Sinclair et al., 2005). Indeed, there is evidence that limited TR at high VPD levels leads to water conservation and enhanced drought tolerance in wheat and soybean (Hufstetler et al., 2007; Schoppach et al., 2014). Similarly, substantial genetic variability in early transpiration decrease at a high threshold of soil water content exists in soybean (Hufstetler et al., 2007), wheat (Faralli, Williams, et al., 2019; Schoppach & Sadok, 2012) and pearl millet (Kholová et al., 2010). Early stomatal closure behaviour has been often linked to biomass and yield maintenance in several species, suggesting that, under fluctuating watering regimes, conservative responses might be considered a preferable trait for crop improvement. These traits along with an extensive phenotypic characterization have never been explored in a large *A. donax* panel and compared with more stress‐tolerant species such as *A. donaciformis* and *A*. *plinii*. Some information is available for shoot characterization under environmental stresses (i.e. heat and water stress) in both greenhouse (Ahrar et al., 2017) and field conditions (Haworth, Cosentino, et al., 2017) but only for a limited amount of accessions. This contrasts with the large amount of information available for food and other biomass crops (e.g. miscanthus, wheat, barley, maize).

Therefore, the objectives of this work are to evaluate (1) the degree of interspecific and (2) intraspecific variation for gas‐exchange traits, biomass accumulation, transpiration sensitivity to water stress and VPD as well as biomass dry weight and (3) assess the preferable combination of the above traits to allow fast phenotyping of *A. donax* ecotypes with non‐invasive phenotyping approaches. In addition, we aim to (4) detect traits in *A*. *plinii* and *A. donaciformis* that can be used as targets to select *A. donax* accessions with higher productivity under drought‐prone rain‐fed marginal lands.

## 2 MATERIALS AND METHODS

### 2.1 Sampling, cuttings production and plant material

The species and accessions collected along with their geographical distribution and date of sampling are summarized in File S1. Annual mean precipitation and temperature for each sampling site based on the Italian National Institute of Statistics data (average for each province) are shown in File S2. Briefly, main stems with secondary shoots already developed were sampled for each population and cuttings were produced in loco to avoid tissue dehydration and immediately immersed in water. A minimum number of 80 similar‐sized cuttings from each population was then moved to Fondazione Edmund Mach (FEM) and immediately placed in hydroponic conditions to stimulate root growth. Water was kept at node level and cuttings were maintained at 100 μmol m^−2^ s^−1^ light and an ambient temperature of ~20°C.

By 18 December 2019, a sufficient number of cuttings had produced a primary and lateral root system (*n* = 30–40 per population) and they were immediately transplanted into pots (10 × 10 × 20 cm) containing a similar amount of growing substrate (~600 g of Flora gard special mixture). The pots were then watered to saturation and moved to an environmental controlled growing cabinet to let the plants overcome transplant stress. Light was provided at ~100 μmol m^−2^ s^−1^, temperature ~22°C and relative humidity ~70%. Photoperiod was 14/10 day/night. Fertilizer was applied on 10 January (5 g of organic NPK per pot).

On 18 January 2020, plants (variable number between *n* = 18 and *n* = 30) were selected and taken out from pot while maintaining soil and intact root systems. The plants were enclosed in bags and Styrofoam boxes and immediately shipped to the National Plant Phenomics Centre (NPPC) in Aberystwyth with a next day flight delivery under controlled conditions The remaining plants (between *n* = 6 and *n* = 10) were kept at the FEM. Plants at the NPPC were immediately transplanted in round pots (23 × 17 × 20.5 cm) all containing 2300 g Levington F2 substrate. Subsequently, plants were placed under controlled environmental conditions (~100 μmol m^−2^ s^−1^, temperature ~22°C and relative humidity ~90%) for recovery from transplant and shipment. The light level was increased to 300 μmol m^−2^ s^−1^ and RH was decreased to 60% on the 2nd of March after full plant recovery.

### 2.2 Experiment 1: Physiological characterization under optimal environmental conditions

A variable number of plants per population (*n* = 5–12) were grown in a greenhouse at FEM under natural light, temperature‐controlled conditions (18°C average and 60% relative humidity). Plants were arranged in a randomized design in blocks (*n* = 5–12). Starting from the 7th leaf stage, plants were subjected to a series of physiological characterizations. All the measurements were carried out between January and February 2020.

### 2.3 Gas‐exchange analysis

Photosynthesis measurements were performed on the middle of the leaf lamina between 9:00 and 14:00 on the most fully expanded leaf for all the accessions (leaf number variable from 7th to 9th) (*n* = 5). Measurements of the response of CO_2_ assimilation rate per unit of leaf area (*A*) to sub‐stomatal CO_2_ concentrations (*C_i_*) were performed in the middle of the tagged leaf using an open infrared gas‐exchange system and a 2 cm^2^ leaf cuvette with an integral blue–red LED light source (LI‐6400–40; LI‐COR). In the cuvette, PPFD was maintained at a saturating level of 1500 µmol m^−2^ s^−1^, a leaf temperature of 30 ± 0.1°C, a VPD between 1.2 and 1.5 kPa and a ambient CO_2_ concentration (*C_a_*) of 400 µmol mol^−1^. When steady‐state conditions were achieved, *C_a_* was sequentially decreased to 300, 200, 150, 75 and 50 µmol mol^−1^ before returning to the initial concentration of 400 µmol mol^−1^. This was followed by a sequential increase to 500, 700, 900, 1100, 1300 and 1500 µmol mol^−1^. Readings were recorded when *A* had stabilized to the new conditions. The maximum velocity of Rubisco for carboxylation (*V*_cmax_) and the maximum rate of electron transport demand for ribulose 1,5‐bisphosphate (RuBP) regeneration (*J*_max_) were derived by curve fitting while *A_sat_* (*A* at 400 µmol mol^−1^ and saturating light) and *g_s_* (stomatal conductance at 400 µmol mol^−1^ and saturating light) are logged values. The same leaves were then subjected to step changes in light analysis but only for a limited number of accessions (*A. donax* Bersezio, Sesto Fiorentino, Torviscosa—*A. donaciformis* Marina di Andora—*A*. *plinii* Castel Maggiore). All measurements were collected between 8:30 and 15:00 and randomized to avoid any potential diurnal influence over a 6‐week measurement period. Prior to measurement, leaves were first equilibrated at a PPFD of 100 µmol m^−2^ s^−1^ until both *A* and stomatal conductance (*g*_s_) reached ‘steady state,’ defined as a ∼2% maximum change in rate during a 10‐min period (generally 60 min). After equilibration, PPFD was increased to 1500 µmol m^−2^ s^−1^ for 1 h, and subsequently returned to 100 µmol m^−2 ^s ^−1^ for 1 h. The conditions inside the leaf cuvette were kept constant at 20 ± 0.1°C leaf temperature, at VPD of ∼1–1.4 kPa and at 400 µmol CO_2_ mol^−1^ air (ambient CO_2_ concentration, *C_a_*).

### 2.4 Stomatal anatomical features

The same leaves as used for gas‐exchange analysis were subjected to stomatal assessment. Stomatal impressions were collected at the same point of the leaf lamina used for gas‐exchange analyses, on both the adaxial (*n* = 6) and abaxial (*n* = 6) side of the leaf. An impression was made using nail polish and, after the material had dried, adhesive tape was used to place it on a microscope slide. Stomatal density (SD) and pore length (PL) were determined using a light microscope by averaging the value of two fields of view for each leaf with a size of ∼1000 µm^2^ captured from each impression.

### 2.5 Experiment 2: Transpiration sensitivity to VPD

For TR (mg H_2_O m^−2^ s^−1^) response to VPD (kPa) analysis, a bespoke system was developed allowing the application of several VPD steps. A modified growth chamber (Sanyo Incubator; Sanyo, Moriguchi) was used. A light system was installed in the chamber providing ∼250 µmol m^−2^ s^−1^ PPFD at plant height. A humidity sensor and a thermocouple were installed inside the chamber and positioned at plant height and air temperature and relative humidity were recorded every minute. Measurements were performed on two consecutive days and on a subset of accessions (*A. donax* Bologna, Torviscosa, Bersezio, Zambana, Sesto Fiorentino—*A. donaciformis* Marina di Andora—*A*. *plinii* Castel Maggiore). Three VPD levels were targeted (1, 1.5, 2.5 and 3.5 kPa) on both days and applied sequentially for 60 min after an equilibration period of 30 min. Variation in VPD was achieved by drying entering air in the chamber with silica gel to balance the humidifying effect of the transpiring leaves (i.e. from 70% to 20% RH for highest VPD levels). Pots, previously enclosed in a plastic bag to limit soil evaporation, were weighed with a balance with a resolution of 0.1 g (Kern, PLS). At the end of the VPD treatment period, pots were weighed again and the rate of mass change attributed to TR was calculated. The corresponding VPD values were calculated from the temperature and relative humidity data and averaged for the measurement period. Following TR measurements on the second day, leaf area of each plant was determined so the TR data for plants could be normalized relative to leaf area and time. Leaf area was assessed non‐destructively as leaf width × leaf length × *k*, where *k* = 0.858 is the shape factor commonly used for Poaceae (Gioia et al., 2015). In File S3, an example of a daily VPD cycle is shown.

### 2.6 Experiment 3: High‐throughput phenotyping under reduced soil water availability

After full recovery from shipment and a period of growth in a controlled environment greenhouse, the plants were moved to the LemnaTec automated system of the NPPC on 28 April 2020. Pots were automatically watered daily to a set target weight of ~3750 g (~2500 ml of available water content (AWC) and a volumetric water content of ~45%). During the experiment, plants were grown at ~25/20°C day/night temperature on average and ~60% of relative humidity with an average daily photon flux density of 400 µmol photons m^−2^ s^−1^ from natural daylight supplemented by white LED grow lights (KP‐4 full spectrum; Kroptec) (14‐h/10‐h light–dark photoperiod) system; in File S4, the average daily values are shown. A liquid feed (Chempak No. 2 25:15:15 NPK, 500 ml plant^−1^, Thompson and Morgan) was applied on 26 April to all the pots. The experiment was arranged in a unbalanced randomized block 3 × 6/3 × 2 factorial design with factor ‘Species’ having three levels (*A. donax*, *A. donaciformis* and *A*. *plinii*), factor ‘Accession’ having either three or six levels (see sampling section) and factor ‘Watering’ having two levels (well‐watered, [WW] and water‐stressed [WS]) in nine blocks (*n* = 3–9 for each treatment).

### 2.7 Stress application and recovery period

The progressive soil drying treatment started on 4 May 2020 by removing watering to the selected water stress pots (WS) and recorded as ‘days after treatment’ 0 (DAT 0). WW pots were maintained at ~3750 g throughout the experiment. Pot weight and re‐watering (WW only) were recorded in the evening (~19:30–21:00) and in the morning (~4:30–6:00). Due to the significant differences in whole plant water‐use between *A. donax*, *A. donaciformis* and *A*. *plinii*, the length of the stress application was 16 days for *A. donax* (15 May) and 22 days for *A. donaciformis* and *A*. *plinii* (26 May) followed by a recovery period to pot capacity of 7 days.

Water content in the pot was then expressed as the fraction of transpirable soil water (FTSW). Total transpirable soil water (TTSW) was calculated as the difference between the pot at 100% AWC and when the transpiration of the plants was ~10% of the control plants. The FTSW value for each DAT was then calculated as FTSW = (WTn − WTf)/TTSW and WTn represents the pot weight on a given DAT and WTf the pot weight of a stressed plant showing ~10% of the transpiration of the control plants. In File S5, the dynamics of FTSW for WW and WS are shown for each species.

### 2.8 Gravimetric assessment of water use and sensitivity to soil drying

Daytime water use (WU_day_) and nighttime water use (WU_night_) were estimated by subtracting mean water use from soil‐only pots (*n* = 25 randomly placed in the experiment) to whole pot water loss. Recovery was calculated as the ratio between WU after 6 days of recovery and the average WU before the stress period and expressed as %. The data of the experiment were analysed by plotting WU (or TR) against FTSW as shown in Faralli, Williams, et al. (2019) and Sadok and Tamang (2019). Given the shape of the TR response to FTSW observed for most genotypes, the data were subjected to a segmented regression analysis. The applied model is as follows:

Y1=slope1×X+intercept1,

YatX0=slope1×X0+intercept1,

Y2=YatX0+slope2×(X‐X0),

Y=If(X<X0,Y1,Y2),

where Intercept1 is the *Y* value where the first line segment intersects the *Y*‐axis (*Y*‐intercept), slope1 is the slope of the first line segment (slope1, slope of the curve after stomatal closure) and slope2 is the slope of the second line segment (slope2, slope of the curve before stomatal closure). *X*0 is the *X* value where the two line segments intersect (WU_bp_, FTSW value at which WU starts to decrease under reduced water availability). Data from VPD experiment were analysed with the same segmented model as described above.

### 2.9 RGB imaging for dynamic projected leaf area and senescence

Plants were imaged every day between 24:00 and 4:00 h using a LemnaTec Scanalyzer phenotyping platform (LemnaTec GmbH). Pots were automatically conveyed into an imaging chamber equipped with a visible light (RGB) sensor with a 2454 × 2056 pixel resolution. Six side views (SV) images were taken at 0°, 15°, 30°, 45°, 60°, 75° and 90°. Image analyses were performed using a bespoke R‐based pipeline for image segmentation. Projected leaf area was estimated as the green pixels in the SV with greatest area (i.e. the maximum area of the plant). The green:red ratio was then calculated for each area decile by height in each segmented image, and the value was used as a proxy of leaf senescence to assess dynamic loss of green pixels during the stress period (Cai et al., 2016). Senescence was then expressed in WS plants as % of WW plants.

#### 2.9.1 Non‐destructive versus destructive leaf area estimation

A second set of plants (*n* = 10) were grown in the same conditions as above and used for destructive harvest during the experimental period. Plants were harvested at different growth phases and leaf number, total leaf area, leaf and stem dry weight were recorded. Correlation between destructive and imaging‐based leaf area was carried out to evaluate the strength of image‐based dataset.

#### 2.9.2 Plant harvest

On 20 May (*A. donax*) and 2 June 2020 (*A. donaciformis and A*. *plinii*), plants were hand harvested. Initially, plant height at the top ligule and top leaf were recorded, followed by leaf number. Fresh weight was determined for leaf and stem, separately; after oven drying at 80°C for 4 days, leaf dry weight, stem dry weight and total dry weight were recorded.

#### 2.9.3 Statistical analysis

All the data were analysed with Rstudio (RStudio Team 2015) and GraphPad Prism 4.0. Curve fitting and parameters estimation for *A*/*C_i_* curves were assessed with the ‘Plantecophys’ package (Duursma, 2015). Stomatal and photosynthetic induction data were analysed according to the exponential model of Vialet‐Chabrand et al. (2013) as described in Faralli, Cockram, et al. (2019) and McAusland et al. (2016). TR sensitivity to VPD data were analysed with piecewise modelling according to Sadok and Tamang (2019). Principal component analysis was carried out with ‘ggbiplot’ package. All the data were then checked for normality and homogeneity of variance and subjected to one‐, two‐ or unbalanced three‐way ANOVA depending on factors level. Block effect was included in the analysis as well when present. Fisher's test was used for means separation.

## 3 RESULTS

### 3.1 Experiment 1

#### 3.1.1 Photosynthetic capacity and stomatal anatomical features

A significant interspecific variation was observed for *A*_sat_ and *g*_s_ (*p* < 0.001) with *A*. *plinii* showing higher *A*_sat_ (25.5 µmol m^−2 ^s^−1^) and *g*_s_ (0.41 mol m^−2^ s^−1^) values by 20% on average compared to *A. donaciformis* and *A. donax* (Table 1). On the contrary, a significant intraspecific variation was not detected (*p* = 0.809; *p* = 0.601, respectively) for *A*_sat_ and *g*_s_. Species and Accession factors were not significant for iWUE, although a trend (*p* = 0.082) was recorded for Species. Intraspecific and interspecific variation for ETR was evident with *A*. *plinii* and *A. donax* showing higher ETR values than *A. donaciformis* (*p* < 0.05).

**TABLE 1:** Summary of *A*/*C* *_i_* curves output for all the species and accessions. Average values are shown for *in vivo* Rubisco carboxylation velocity (*V*_cmax_), maximum electron transport chain capacity for RuBP regeneration (*J*_max_), CO_2_ assimilation rate at saturating light (*A*_sat_), CO_2_ assimilation rate at saturating light and elevated CO_2_ (*A*_max_), electron transport rate (ETR) and intrinsic water‐use efficiency (*_i_* *WUE*). Average stomatal density and pore length are shown as well. ANOVA output is shown in the table while different letters represent significant differences according to Fisher's test

Species	Accession		*V*_cmax_ (µmol m^−2^ s^−1^)	*J*_max_ (µmol m^−2^ s^−1^)	*A*_sat_ (µmol m^−2^ s^−1^)	*A*_max_ (µmol m^−2^ s^−1^)	ETR (µmol m^−2^ s^−1^)	*g*_s_ (mol m^−2^ s^−1^)	_i_WUE (µmol mol^−1^)	Stomatal density (mm^2^)	Average stomatal length (µm)
*Arundo donaciformis*	Andora		61.83^a^	182.40^bc^	20.46^a^	39.66^c^	123.10^abc^	0.26^a^	80.56^a^	96.5	15.15
	Cervo		66.33^abc^	194.26^c^	20.17^a^	40.16^c^	106.29^a^	0.26^a^	87.64^a^	65.1	20.28
	Finale		61.09^a^	167.62^abc^	20.10^a^	36.65^c^	123.93^abc^	0.26^a^	80.81^a^	96.6	19.55
*Arundo donax*	Bersezio		70.49^bcd^	157.90^ab^	24.23^bc^	35.65^bc^	145.14^cd^	0.39^bcd^	63.02^a^	81.9	26.51
	Bologna		73.22^bcd^	156.99^ab^	22.14^ab^	34.76^bc^	147.87^d^	0.31^abc^	77.20^a^	75.5	26.47
	Finale Ligure		60.94^a^	137.51^a^	18.69^a^	31.86^b^	115.53^ab^	0.25^a^	81.57^a^	84.0	25.77
	Sesto Fiorentino		64.48^ab^	144.78^a^	21.48^ab^	32.97^b^	130.92^bcd^	0.31^abc^	74.58^a^	71.1	25.85
	Torviscosa		66.14^ab^	152.89^a^	22.27^ab^	34.57^bc^	140.11^cd^	0.35^bcd^	66.21^a^	81.1	24.58
	Zambana		62.24^a^	145.73^a^	20.24^a^	29.69^a^	112.31^ab^	0.28^ab^	75.91^a^	103.9	22.99
*Arundo plinii*	Argelato		81.76^d^	247.73^d^	26.86^c^	51.15^d^	129.63^bcd^	0.39^cd^	70.78^a^	105.4	16.76
	Castel Maggiore		78.47^bcd^	186.10^bc^	24.71b^c^	40.45^c^	125.14^abc^	0.43^d^	59.56^a^	92.4	19.02
	Sasso Marconi		80.13^cd^	229.62^d^	26.48^c^	48.62^d^	134.35^bcd^	0.40 ^cd^	71.84^a^	94.8	16.48
		SEM	4.94	10.59	1.29	2.05	7.09	0.04	8.07	3.38	0.50
		df (one‐way)	11.00	11.00	11.00	11.00	11.00	11.00	11.00	11	11
		Species *p*	<0.001	<0.001	<0.001	<0.001	0.047	<0.001	ns	<0.001	<0.001
		Accession *p*	ns	0.017	ns	0.022	0.024	ns	ns	<0.001	<0.001

*In vivo* Rubisco carboxylation velocity (*V_cmax_*) and maximum electron transport chain capacity for RuBP regeneration (*J_max_*) were significantly (*p* < 0.001) higher in *A*. *plinii* than *A. donaciformis* than most of the *A. donax* accessions (Table 1; File S6). Indeed, a significant intraspecific variation (*p* = 0.017) was recorded for *J*_max_.

Intraspecific and interspecific variation (*p* < 0.001 for both) was observed for SD with *A*. *plinii* showing the highest SD (Table 1). Similarly, there was species variation for PL as well as variation between accessions (*p* < 0.001). A negative correlation was observed overall between SD and PL with *A. donax* showing the largest intraspecific variation for the relationship SD/PL (data not shown).

#### 3.1.2 Gas‐exchange response to dynamic light

In general, while most of the traits estimated were not significantly different, either intraspecifically or interspecifically, a significant variation was observed for the time for stomatal opening (*K_i_*) between species (*p* = 0.011) (Table 2; File S7). Indeed, *A*. *plinii* showed slow stomatal opening while *A. donaciformis* and *A. donax* were faster than *A*. *plinii*, in particular the Bersezio and Marina di Andora accessions with a time constant for stomatal opening of 3.4 minutes on average.

**TABLE 2:** Time constant for stomatal opening (*K* *_i_*) and closing (*K* *_d_*) and time to reach 95% *A* for five accessions. Data were estimated from step changes protocols using an exponential model. ANOVA output is shown in the table while different letters represent significant differences according to Fisher's test. Data shown are means (*n* = 5–6)

Species	Accession	*K_i_* (minutes)	*K_d_* (minutes)	Time to reach 95% *A*
*Arundo donax*	Bersezio	3.33^a^	6.05^a^	6.85^a^
*A. donax*	Sesto Fiorentino	6.05^b^	7.48^a^	6.09^a^
*A. donax*	Torviscosa	6.78^b^	4.96^a^	7.56^a^
*Arundo donaciformis*	Marina di Andora	3.54^a^	6.02^a^	8.31^a^
*Arundo plinii*	Castel Maggiore	9.74^c^	5.11^a^	7.43^a^
SEM		1.14	1.20	0.88
*df*		4	4	4
*n*		6	6	6
Species *p* value		0.011	ns	ns
Accession *p* value		ns	ns	ns

### 3.2 Experiment 2

#### 3.2.1 Transpiration sensitivity to VPD

**FIGURE 1:**
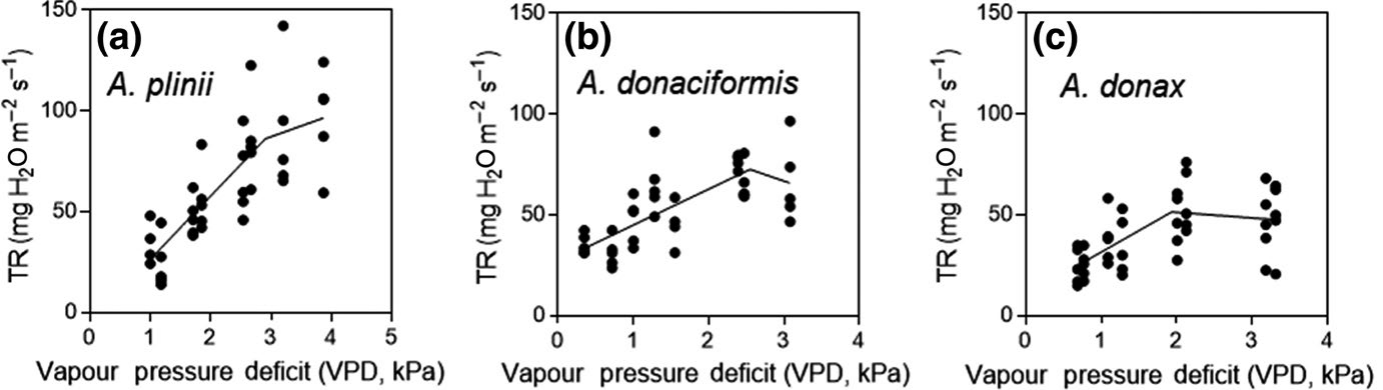
Diversity in whole‐plant transpiration rate (TR) responses to increasing vapour pressure deficit (VPD) among *Arundo* accessions. All the accessions showed a segmented response, although the sensitivity to VPD was much larger in *Arundo donax* Zambana (c) than *Arundo donaciformis* Marina di Andora (b) and in particular *Arundo plinii* Castel Maggiore (a). For statistics and curve fitting, please see Table 3

Most of the accessions tested started to restrict transpiration between 1.5 and 3 kPa VPD. *A*. *plinii* showed the most non‐conservative response (TR_bp_ at 2.97 kPa) while the most conservative accession was *A. donax* Sesto Fiorentino (TR_bp_ at 1.52 kPa, *p* < 0.001 among species; Table 3; Figure 1). There was an intraspecific variation for TR_bp_ in *A. donax*, with some accessions (Zambana, Sesto Fiorentino) showing a more conservative response to VPD than others (Bersezio, Torviscosa and Bologna).

**TABLE 3:** Summary statistics of the regression fits of whole‐plant transpiration rate (TR) response curves to increased atmospheric vapour pressure deficit in seven *Arundo* accessions. ANOVA output is shown in the table while different letters represent significant differences according to Fisher's test. Data shown are means (*n* = 5–6)

Species	Accession	Intercept_1_	Slope_1_	TR_bp_ (kPa)	Slope_2_	*R* ^2^
*Arundo donaciformis*	Marina di Andora	27.08	17.82	2.52	−22.11	0.69
*Arundo plinii*	Castel Maggiore	−5.19	31.43	2.97	4.73	0.95
*Arundo donax*	Bologna	0.16	12.45	2.16	2.08	0.88
*A. donax*	Torviscosa	2.90	27.49	2.19	−17.89	0.90
*A. donax*	Bersezio	3.26	20.48	2.25	−4.41	0.78
*A. donax*	Zambana	10.08	21.35	1.93	−2.79	0.89
*A. donax*	Sesto Fiorentino	−0.34	33.52	1.52	12.92	0.68
Species *p* value		0.012*	0.881	<0.001***	0.536	
Accession *p* value		0.048*	0.051	0.012*	0.195	

* *p* < 0.05.

*** *p* < 0.001.

### 3.3 Experiment 3

#### 3.3.1 Plant transpiration and water‐use under WW conditions

Under WW conditions, a significant interspecific variation was found (*p* < 0.001) for plant daytime WU on average, with *A. donax* having the highest daytime WU (252.8 g day^−1^ on average) and *A*. *plinii* showing the lowest values (166.1 g day^−1^ on average; Table 4). Similarly, nighttime WU varied among species (*p* < 0.001) with *A. donax* using 33.5 g day^−1^ of water on average, *A. donaciformis* 26.7 g day^−1^ and *A*. *plinii* 24.2 g day^−1^ (Table 5). Indeed, the ratio between daytime and nighttime was significantly higher (*p* = 0.040) in *A*. *plinii* than the other two selected species. Intraspecific variation was present for both traits (daytime and nighttime WU, *p* < 0.001) and within all the species (*p* < 0.001). The largest variation was present in *A. donax* where Finale Ligure and Sesto Fiorentino accessions had a significantly (*p* < 0.001) lower daytime and nighttime WU than largely non‐conservative accessions such as Bersezio and Zambana.

**TABLE 4:** Daytime and nighttime water use (WU) for well‐watered plants. Data were collected over 22 days for *Arundo donax* and 28 days for *Arundo plinii* and *Arundo donaciformis* with a high‐throughput automated weighing platform. ANOVA output is shown in the table while different letters represent significant differences according to Fisher's test. Data shown are means (*n* = 3–9).

Species	Accession	Daytime WU (g day^−1^)	Nighttime WU (g day^−1^)
*A. donax*	Bologna	270.7 i	33.7 f
*A. donax*	Finale Ligure	220.0 f	31.9 ef
*A. donax*	Torviscosa	255.7 gh	31.0 def
*A. donax*	Bersezio	311.5 j	37.0 g
*A. donax*	Zamabana	265.7 hi	33.7 f
*A. donax*	Sesto Fiorentino	193.2 de	32.4 ef
*A. donaciformis*	Finale Ligure	171.1 bc	24.1 ab
*A. donaciformis*	Andora	248.5 g	29.5 cde
*A. donaciformis*	Punta Cervo	202.5 ef	26.7 abcd
*A. plinii*	Castel Maggiore	146.1 a	22.4 a
*A. plinii*	Argelato	168.2 b	23.4 a
*A. plinii*	Sasso Marconi	183.8 cd	26.8 bc
	SEM	5.5	1.2
	Species *p* value	<0.001	<0.001
	Accession *p* value	<0.001	<0.001

**TABLE 5:** Dry weight biomass (whole plant, stem and leaf), leaf number and plant height collected at the end of the experiment for both WW and WS plants. Asterisks in WS show significant differences compared to the relative WW according to one‐way ANOVA

Species	Accession		Watering	Plant height (cm)	Leaf number	Leaf dry weight (g)	Stem dry weight (g)	Whole‐plant dry weight (g)
*Arundo donaciformis*	Andora		WW	187.50	20.25	6.73	3.93	10.65
			WS	162.00*	17.75*	5.05*	2.70	7.75*
	Finale Ligure		WW	130.60	13.00	8.74	7.72	16.46
			WS	139.00	12.20	7.76	4.70*	12.46*
	Punta Cervo		WW	155.00	14.00	9.60	5.80	15.40
			WS	120.67*	12.00*	6.20*	3.73*	9.93*
*Arundo donax*	Bersezio		WW	101.22	16.00	13.20	5.54	18.74
			WS	77.44*	13.78*	9.33*	3.60*	12.93*
	Bologna		WW	120.78	16.78	13.19	6.51	19.70
			WS	102.56*	13.33*	9.77*	5.26	15.02*
	Finale Ligure		WW	103.25	14.50	9.48	3.85	13.33
			WS	85.25*	12.50*	8.10	3.85	11.95
	Sesto Fiorentino		WW	99.17	13.83	9.63	5.27	14.90
			WS	90.17	13.17	8.27	4.10	12.37
	Torviscosa		WW	90.63	15.50	11.21	5.11	16.33
			WS	73.00*	12.13*	8.46*	3.46*	11.93*
	Zambana		WW	112.14	15.86	12.26	6.56	18.81
			WS	97.88*	12.88*	10.16*	5.33	15.49*
*Arundo plinii*	Argelato		WW	114.60	13.80	5.52	3.38	8.90
			WS	116.20	14.40	5.10	3.38	8.48
	Castel Maggiore		WW	107.00	16.00	6.45	5.80	12.25
			WS	108.00	12.33*	4.97*	3.53	8.50*
	Sasso Marconi		WW	134.17	14.33	7.30	4.48	11.78
			WS	120.17	13.50	5.98*	3.75	9.73
		SEM		8.38	0.73	0.76	0.74	1.41
		Species *p*		<0.001	ns	<0.001	0.007	<0.001
		Accession *p*		<0.001	<0.001	<0.001	<0.001	<0.001
		Watering *p*		<0.001	<0.001	<0.001	<0.001	<0.001
		Species x Watering *p*		ns	0.016	0.039	ns	ns
		Accession x Watering *p*		ns	ns	ns	ns	ns

Abbreviations: WS, water‐stressed; WW, well‐watered.

#### 3.3.2 Transpiration sensitivity to reduced water availability

**FIGURE 2:**
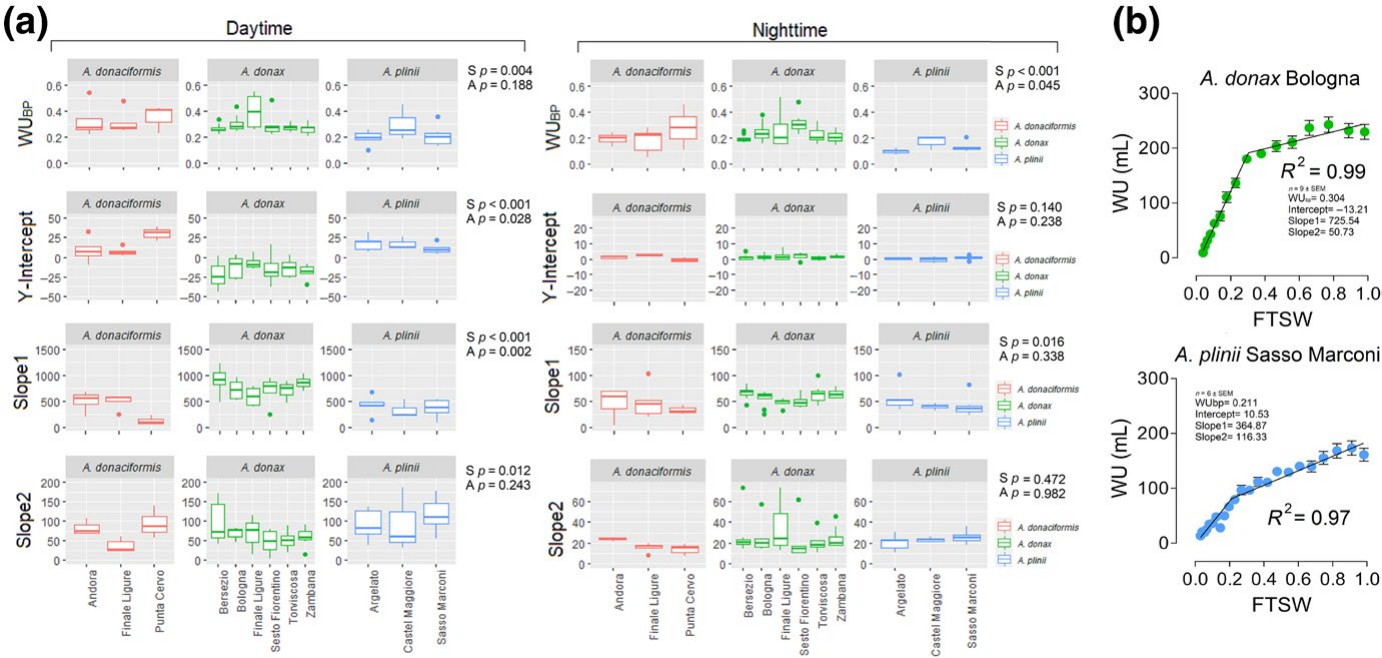
Trait estimated with piecewise modelling in all the species and accessions (a) and example of water‐use dynamics under progressive soil drying conditions (WS, b). Data are shown for all the species and accession (*n* = 3–9) and ANOVA output is shown in Figure (a). FTSW, fraction of transpirable soil water; WS, water‐stressed; WW, well‐watered

The response of WU to FTSW was well described by a segmented regression (R^2^=0.90 on average, Figure 2B). There was a significant interspecific variation for all the estimated traits from piecewise modelling (*p* < 0.001) and for the response of daytime WU to FTSW (Figure 2A).

On the contrary, the response of nighttime WU to FTSW showed a significant interspecific variation for WU_bp_ (*p* < 0.001) and Slope1 (*p* = 0.016). Within each species, there was a significant variation for Y‐intercept (*p* = 0.028) and Slope1 (*p* = 0.002) when daytime WU and FTSW were plotted (Figure 2A). Conversely, there was a significantly intraspecific variation in WU_bp_ (*p* = 0.045) and for nighttime WU versus FTSW. There was a significant interspecific variation for recovery after stress (*p* < 0.001), with *A*. *plinii* capable at having a full WU recovery from stress while *A. donax* and *A. donaciformis* had an average daytime WU recovery of 74.2% and 78.5%, respectively (File 8). Interestingly, no variation was recorded for nighttime WU recovery, with an average 100% recovery for most of the accessions.

#### 3.3.3 Projected leaf area accumulation under reduced water availability

**FIGURE 3:**
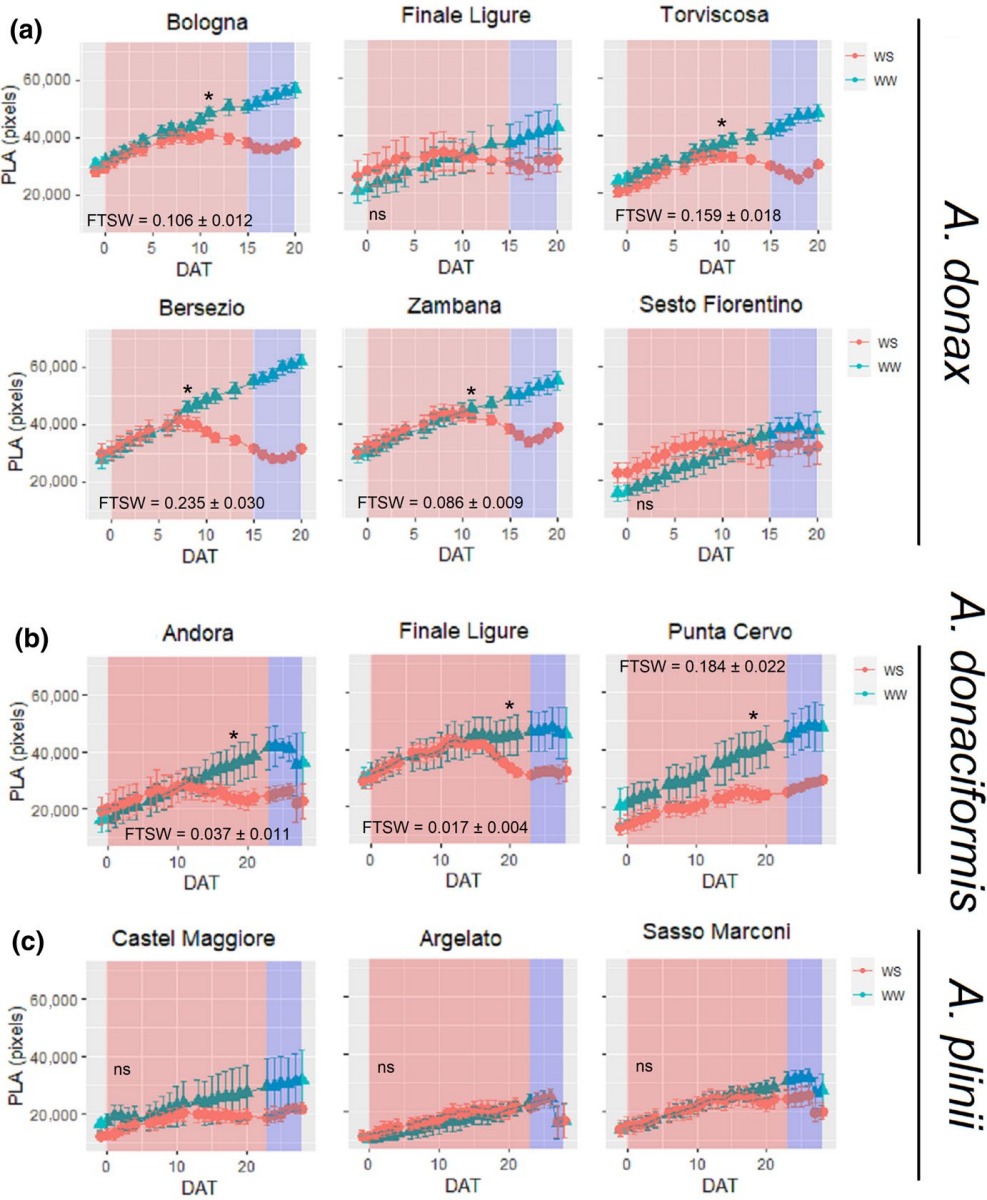
Projected leaf area (PLA, pixels) for *Arundo donax*, *Arundo donaciformis* and *Arundo plinii* accessions grown under well‐watered (WW, blue triangles) and water‐stressed (WS, red circles) conditions. Red background represents the length of the stress application to WS while blue background represents the recovery period. Data are means (*n* = 3–9) ± standard error of the means (SEM). Asterisks represent the day in which WW and WS were significantly different according to one‐way ANOVA and for each accession. The level of FTSW for WS at which there was a significant difference between WS and WW plants is shown in the graph. DAT, days after treatment; FTSW, fraction of transpirable soil water

A significant correlation (*p* = 0.007, *r* ^2^ = 0.61, *y* = 0.0177*x* + 185.21) was observed between projected leaf area (PLA) and leaf area assessed destructively, thus validating the imaged‐based estimation of leaf area dynamics. Under WW conditions, all the accessions showed a linear PLA increase, with *A. donax* accessions having a larger PLA (Figure 3A) than *A*. *plinii* (Figure 3C) and, to a lesser extent, *A. donaciformis* (Figure 3B; *p* < 0.001). A significant intraspecific variation (*p* < 0.001) was observed in all the species. In particular, within *A. donax*, the accessions from Bologna, Torviscosa, Bersezio and Zambana had a significantly higher PLA accumulation throughout the experiment than Finale Ligure and Sesto Fiorentino. The application of WS reduced PLA accumulation in most of accession of *A. donax* and *A. donaciformis* but there was no statistical differences between WW and WS in *A*. *plinii*. Indeed, a significant interaction (*p* < 0.001) was observed between Species and Accession factors with watering regime.

#### 3.3.4 Dynamic leaf senescence under reduced water availability

**FIGURE 4:**
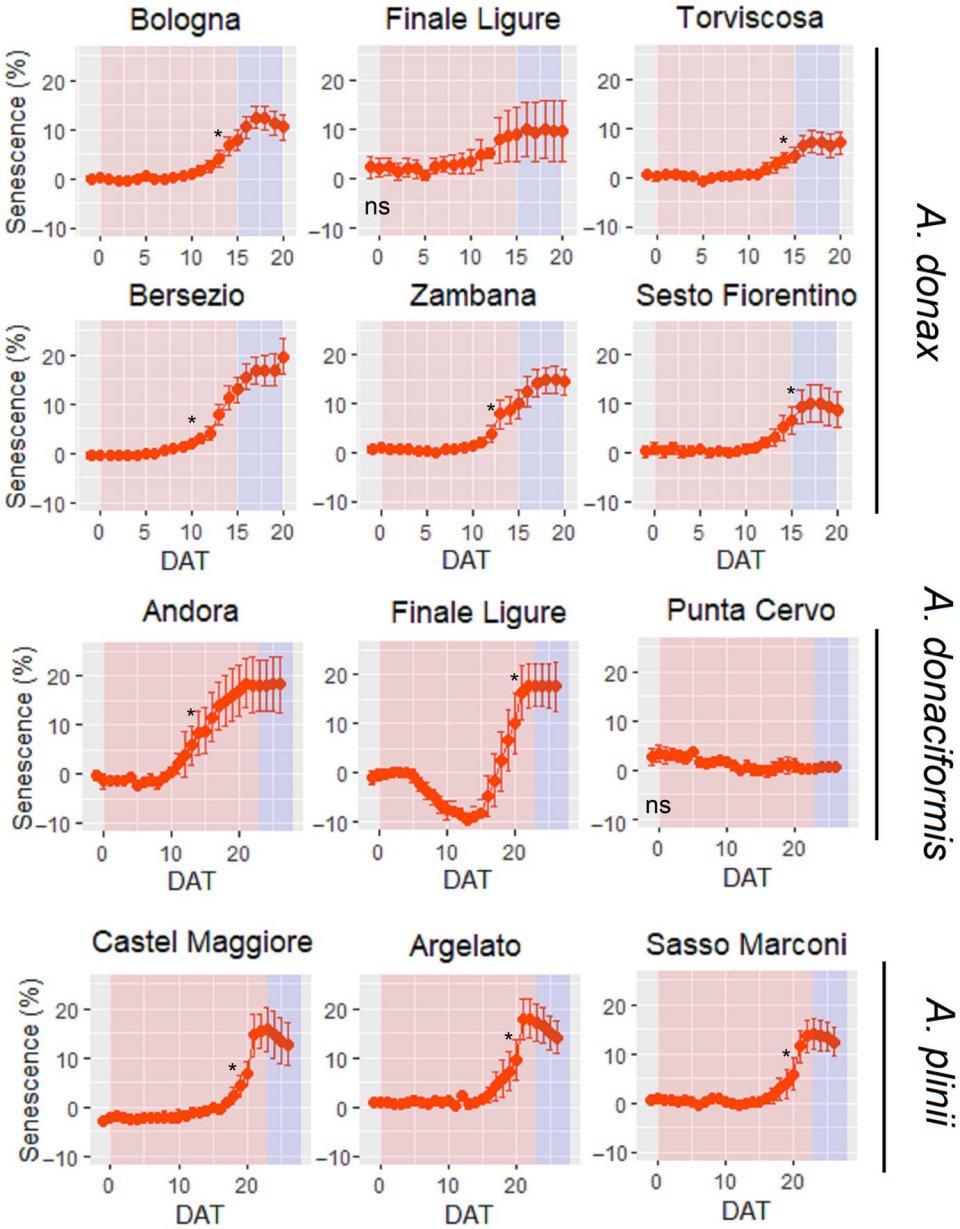
Senescence (%) for WS plants assessed through a green:red pixels ratio from segmented RGB images. Data are shown as % with respect to the relative WW control. Red background represents the length of the stress application to WS while blue background represents the recovery period. Asterisks represent the DAT at which WW and WS were significantly different (i.e. significant increase in senescent area) according to one‐way ANOVA. Data are means (*n* = 3–9) ± standard error of the means (SEM). DAT, days after treatment; WS, water‐stressed; WW, well‐watered

Overall, there was a significant effect of Species, Accession and Watering in senescence (%) (*p* < 0.001). In general, senescence was detected at stronger stress conditions than PLA reductions in *A. donax* as the increase in green:red ratio was significant mostly at the end of the stress treatment (Figure 4). Intraspecific variation for senescence was present in *A. donax* with Finale Ligure showing no onset of senescence while this was present in all the other accessions. In *A. donaciformis*, a significant intraspecific variation was observed for senescence. On the contrary, all *A*. *plinii* accessions showed senesced leaves during the stress application even if PLA was unaffected.

#### 3.3.5 Leaf, stem and total dry weight biomass

Generally, *A. donax* produced a higher DW biomass than *A. donaciformis* and *A*. *plinii* (*p* < 0.001) under WW conditions and significant (*p* < 0.001) variation was present within each species (Table 5). WS reduced biomass DW significantly in most of the accession tested (*p* < 0.001) although the reduction was greater in *A. donaciformis* and *A. donax* than *A*. *plinii*. Very similar patterns were observed for stem and leaf dry weight. There was not a significant effect of Species on leaf number while a significant intraspecific variation for leaf number was present (*p* < 0.001). WS significantly reduced leaf number and plant height in most of the accession (*p* < 0.001), although the reduction was marginal for *A*. *plinii*.

#### 3.3.6 Association analysis and principal components

**FIGURE 5:**
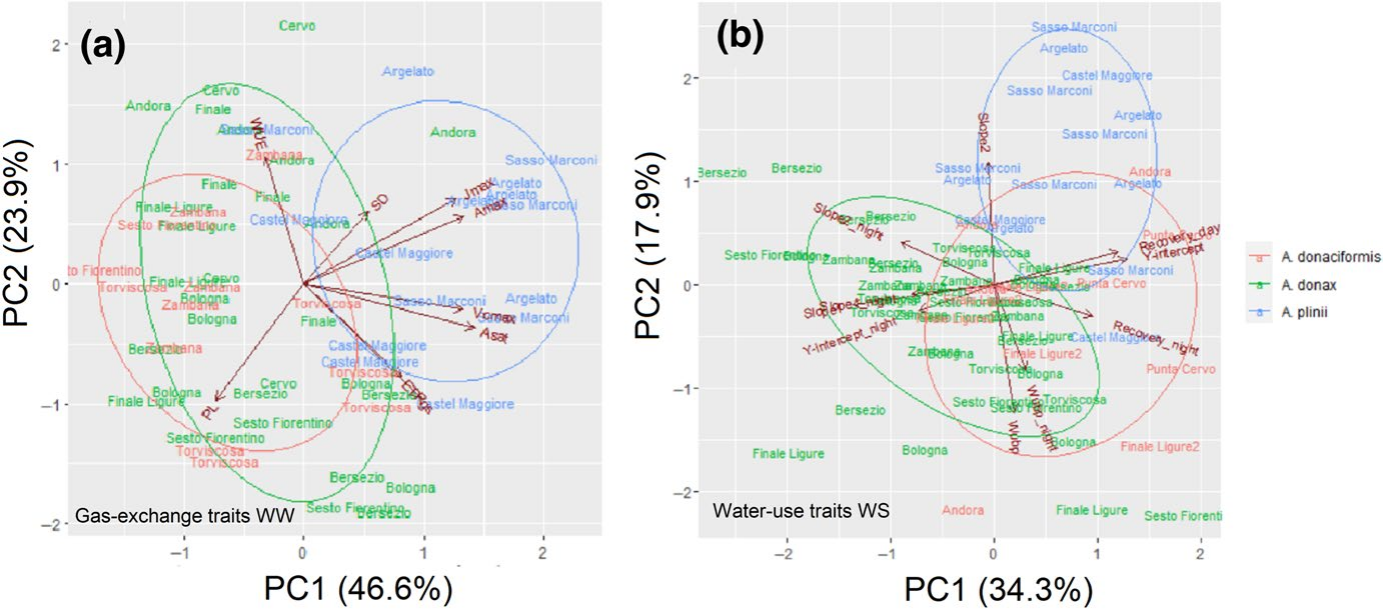
Principal component analysis for (a) gas‐exchange traits for each species under WW conditions and (b) water‐use traits for each species under WS conditions. WS, water‐stressed; WW, well‐watered

Principal components analyses (PCA) were carried out in datasets with balanced replication. In the PCA analysis for gas‐exchange traits (Figure 5A), PC1 and PC2 explained 46.6 and 23.9% of the variation, respectively, and total variance of 70.4%. PC1 loaded positively with SD, *J*_max_, *V*_cmax_ and *A*_sat_, while PC2 loaded positively with _i_WUE. Two main clusters with a significant overlap between *A. donax* and *A. donaciformis* were observed and a distinct cluster for *A*. *plinii*. In the PCA analysis focusing on stress‐response traits (Figure 5B), the three species formed three distinct clusters with PC1 explaining 17.9% of variation and PC2 34.3% (total variance 52.2%). Positive loading for PC1 were WU_bp_ and % of recovery, whereas for PC2 Slope2. *A*. *plinii* and *A. donax* showed the most evident separated clusters with the latter mainly clustering for highly conservative water‐use traits (e.g. WU_bp_).

## 4 DISCUSSION

### 4.1 Interspecific variation is present within the Arundo genus for key adaptive traits

In this work, we present an intraspecific and interspecific physiological assessment for a number of species and accessions belonging to the *Arundo* genus and for key physiological traits never explored so far in these species. In general, the species tested here showed high photosynthetic capacity and intrinsic water‐use efficiency compared with other C_3_, and comparable with C_4_ bioenergy grasses. As in Webster et al. (2016), these species do not achieve high photosynthetic rates through high *g*_s_ (or through lower photorespiratory rates), but rather through an elevated carboxylation velocity of Rubisco and RuBP‐limited photosynthetic rates. Similarly, our work confirms the elevated water requirements in *Arundo* species accompanied by a high SD per unit of leaf area.

The comparative approach we used allowed an assessment for the phenotypic effects of phylogeny versus ploidy‐based relationships among species. In particular, *A. donax* and *A. donaciformis* share the same ploidy level (2*n* = 18*x* = ca. 108; Hardion et al., 2015), while *A*. *plinii* (2*n* = 12*x* = 76; Hardion et al., 2015) and *A. donaciformis* are phylogenetically sister species, being *A. donaciformis* likely derived from *A*. *plinii* following a demiploidization event (Jike et al., 2020). Under unstressed conditions, several of the traits analysed transgressed evolutionary relationship (i.e. phylogenetic distance) and could then be likely ascribed to ploidy levels. The clearest dependence we found was that between productivity‐related traits (plant height, biomass, etc.) and ploidy. These differences are a direct consequence of the well‐known gigas effect, that is, the enlargement of cell and/or organ sizes consequent to increased genome content (Beaulieu et al., 2008; Melaragno et al., 1993; Mowforth & Grime, 1989). Despite the larger biomass of the species with higher ploidy level, large differences exist also between them, with *A. donax* being much more productive in unstressed conditions than *A. donaciformis*. As the extraordinary productivity of *A. donax* may be a consequence of the contribution of the Asian gene pool of species (Jike et al., 2020), further studies comparing eastern and western accessions of *A. donax* invasive clone will be required to disentangle the relative contribution to productivity of ploidy versus genotype of origin.

Another correlation was found in this study between gas‐exchange‐related traits and ploidy, as PCA analysis for gas‐exchange traits revealed two main clusters with a significant overlap between *A. donax* and *A. donaciformis* and a distinct cluster for *A*. *plinii*. In particular, *A*. *plinii* had the highest photosynthetic capacity, *A*_sat_ and *g*_s_, suggesting an elevated investment in gas exchange per unit of leaf area, in line with previous work on these species (Ahrar et al., 2015). These differences are possibly related to the gigas effect, too, as the decreased SD and increased stomatal PL in polyploids have been confirmed to take place in a wide range of angiosperm species (Beaulieu et al., 2008). The physiological consequences of the gigas effect in stomata are usually expected to consist in enhanced gas exchange and, thus, potentially, higher photosynthetic rates (Soltis et al., 2016). However, this prediction is not verified in *Arundo*, where *A*. *plinii* showed higher *g*_s_ and *A*_sat_ than *A. donax* and *A. donaciformis*. This is possibly due to the fact that physiological consequences of polyploidy seem to be largely taxon‐specific, with variable outcomes depending on the relative ratio between photosynthetic rates and number of cells per unit leaf area (Gao et al., 2017; Greer et al., 2018; Vyas et al., 2007; Warner & Edwards, 1993).

Other traits with a more or less markedly transgressive behaviour with regard to evolutionary relationships were the speed of stomatal opening to dynamic light, transpiration sensitivity to VPD and the ratio between daytime and nighttime water‐use. In these cases, however, no direct link with the gigas effect is apparent. Other important factors to be taken into account are also the date of polyploidization and the possible presence of post‐polyploidization adaptation (Soltis et al., 2016). Both polyploidization events in the *Arundo* considered in our study are relatively old, dating back more than 2 million years ago for *A. donaciformis* and even earlier for *A. donax* (Jike et al., 2020), leaving ample space to secondary evolution of the above‐mentioned traits due to post‐speciation adaptation.

Interestingly, a closer trait similarity between the two sister species (*A*. *plinii* and *A. donaciformis*), by contrast, was observed for stress‐response traits. In this case, the only clearly transgressive trait was the daytime capacity of recovery after stress, with *A*. *plinii* able to fully recover *versus* the partial recoveries observed for *A. donax* and *A. donaciformis*. The majority of the drought stress variables considered in the PCA analysis, however, indicated a higher separation of the three species, with the first PCA component separating *A. donax* from *A*. *plinii* and *A. donaciformis* and the second component separating *A. donax* and *A. donaciformis* from *A*. *plinii*. *A*. *plinii* and *A. donax* showed the most evidently separated clusters with the latter mainly clustering for highly conservative water‐use traits. Another common consequence of the gigas effect is the development of larger xylem vessels in polypoids, leading, on the one hand, to higher hydraulic conductivity and possibly increased vulnerability to cavitation, and resulting in polyploids having divergent stress tolerances from their progenitors (Soltis et al., 2016). In our study, *A. donax* showed a significantly higher conservative WU under stress, with an earlier stomatal closure under developing soil moisture deficit than *A. donaciformis* and especially *A*. *plinii*. This is in line with previous observations that different physiological tolerances among individuals with different ploidy levels are mediated not only by stomatal size but also by variation in closure response to the hormone abscisic acid (ABA), which can be modulated by the expression levels of ABA‐responsive genes (Del Pozo & Ramirez‐Parra, 2014). However, also in the case of physiological responses to drought, in species with older ploidy, tolerance to water deprivation is often associated to post‐polyploidization adaptation, although the direction of such adaptation can differ with respect to what we observed in *Arundo* (Maherali et al., 2009).

### 4.2 Intraspecific variation in A. donax and potential high‐throughput phenotyping of favourable traits for selection of superior clones

*Arundo donax* is a clone incapable of sexual reproduction. Molecular analysis of clonal populations collected in America and Europe (Hardion et al., 2012; Hardion, Baumel, et al., 2014; Mariani et al., 2010; Pilu et al., 2014) suggested a minimal intraspecific genetic diversity in *A. donax*. Conversely, analysis of molecular markers and morphology in eight *A. donax* ecotypes from Italy indicated the existence of three distinct genotypes (Pilu et al., 2014) and epigenetic modifications have been hypothesized as an additional source of variation (Guarino et al., 2019). Indeed, in our study, a significant phenotypic variation was observed for several traits in *A. donax*, confirming previous studies where inheritable phenotypic traits such as phenology and leaf functional characters vary even in large collections of European or Italian ecotypes (Ahrar et al., 2017; Fabbrini et al., 2019; Sánchez et al., 2015), especially under stress conditions. For instance, it has been previously shown that the capacity of a Bulgarian *A. donax* ecotype to thrive in harsh environments was linked to an enhanced biosynthesis of isoprenoids in turn leading to a low metabolic impairment under severe stress and rapid recovery (Ahrar et al., 2017). Similarly, in our work, Finale Ligure and Sesto Fiorentino accessions showed a limited biomass dry weight reduction under water stress while this was highly significant in other ecotypes. This limited effect of water stress on biomass was accompanied by several different physiological responses to reduced water availability and VPD. Daytime and nighttime WU were significantly lower in Finale Ligure and Sesto Fiorentino than other accessions and accompanied by an earlier WU_bp_ under reduced water availability. This pronounced stomatal sensitivity to water stress and conservative behaviour, in turn, yielded a higher recovery of WU after stress. In Haworth, Centritto, et al. (2017), the different response of two *A. donax* accessions under drought and recovery was attributed to xylem morphology, with a relatively water‐stress‐tolerant accession showing large xylem vessel diameter, thus facilitating water transport along the stem. It is likely that the potential involvement of xylem plasticity and morphology under reduced water availability may explain some of the variation between *A. donax* accessions found in this work.

In addition, Finale Ligure and Sesto Fiorentino had a lower percentage of senesced leaves compared to the other *A. donax* accessions, potentially driven by the pronounced water‐saving strategy adopted. Leaf senescence can contribute to plant survival under severe water deprivation by enhancing nutrient remobilization to the rest of the plant and limiting transpiration from senesced leaves (Munné‐Bosch & Alegre, 2004). However, a large body of evidence confirms the importance of ‘delayed senescence’ (Rivero et al., 2007) and ‘stay green’ (Borrell et al., 2000) behaviour under stress in crops mainly by providing photosynthetic and yield maintenance. This explains the elevated capacity of WU recovery and biomass maintenance in accessions with reduced senescence such as Finale Ligure, Sesto Fiorentino and, to some extent, Torviscosa.

The diversity in daytime and nighttime water use identified in this work may open up new opportunities for selecting more tolerant *A. donax* ecotypes adapted to a large range of precipitation regimes present in the Mediterranean basin. For instance, our findings confirm that ecotypes with high WU, limited response to VPD and water stress but with a fast growth behaviour would be beneficial in areas where a non‐conservative water‐use strategy is needed to exploit the water available, prevent losses through soil evaporation (e.g. Bersezio, Bologna) and leading to greater biomass production (Ahrar et al., 2017). However, non‐conservative accessions also displayed earlier degrees of senescence, potentially leading to limited advantages under terminal stress conditions. On the contrary, conservative ecotypes such as Finale Ligure and Sesto Fiorentino were characterized by minimal senesce onset and would be suitable for areas where the water‐saving strategy will allow biomass growth even under water‐limited conditions, although lower biomass yield would be expected under optimal growing conditions compared to ‘spender’ accessions. Subsequent studies in field trials will clarify the role of these preferable water‐use traits combinations and the specific ecotype advantage under rain‐fed and water‐limited conditions.

## 5 CONCLUSIONS

In conclusion, we show for the first time the presence of a significant interspecific and intraspecific variation for several important physiological traits in *Arundo* under several environmental conditions. *A*. *plinii* was the most drought‐tolerant species with *A. donax* and *A. donaciformis* being more sensitive. In *A. donax*, a significant intraspecific variation was found with high‐biomass accessions (e.g. Bersezio and Bologna) being very sensitive to water stress following significant reduction in biomass and early onset of senescence. On the contrary, Finale Ligure and Sesto Fiorentino accession had highly conservative water‐use behaviour under water stress and increasing VPD, leading to non‐significant reduction in biomass, limited senescence but limited biomass production under optimal conditions. Additionally, we provide for the first time a series of potential traits to phenotype *A. donax* accession for stress tolerance using non‐invasive imaging approaches (senescence, PLA) and gravimetric assessments (water use and transpiration under water stress and high VPD). In view of the increasing restriction in water availability for crop production, the need to utilize marginal land under rain‐fed conditions and the limitation in conventional breeding in *A. donax*, our work provides novel information on potentially new tolerant ecotypes with combinations of useful traits under different environmental scenarios.

## Supporting information

Supplementary Material

## Data Availability

The data that support the findings of this study are available from the corresponding author upon reasonable request.
