# Supplementary material for: Interspecific and intraspecific phenotypic diversity for drought adaptation in bioenergy Arundo species
Source: Glob Change Biol Bioenergy. 2021 Feb 16;13(4):753–69. doi: 10.1111/gcbb.12810 (PMC7986115; doi:10.1111/gcbb.12810)
Supplement: Supplementary file 1 — Supplementary Material [file GCBB-13-753-s001.pptx]

## Slide 1
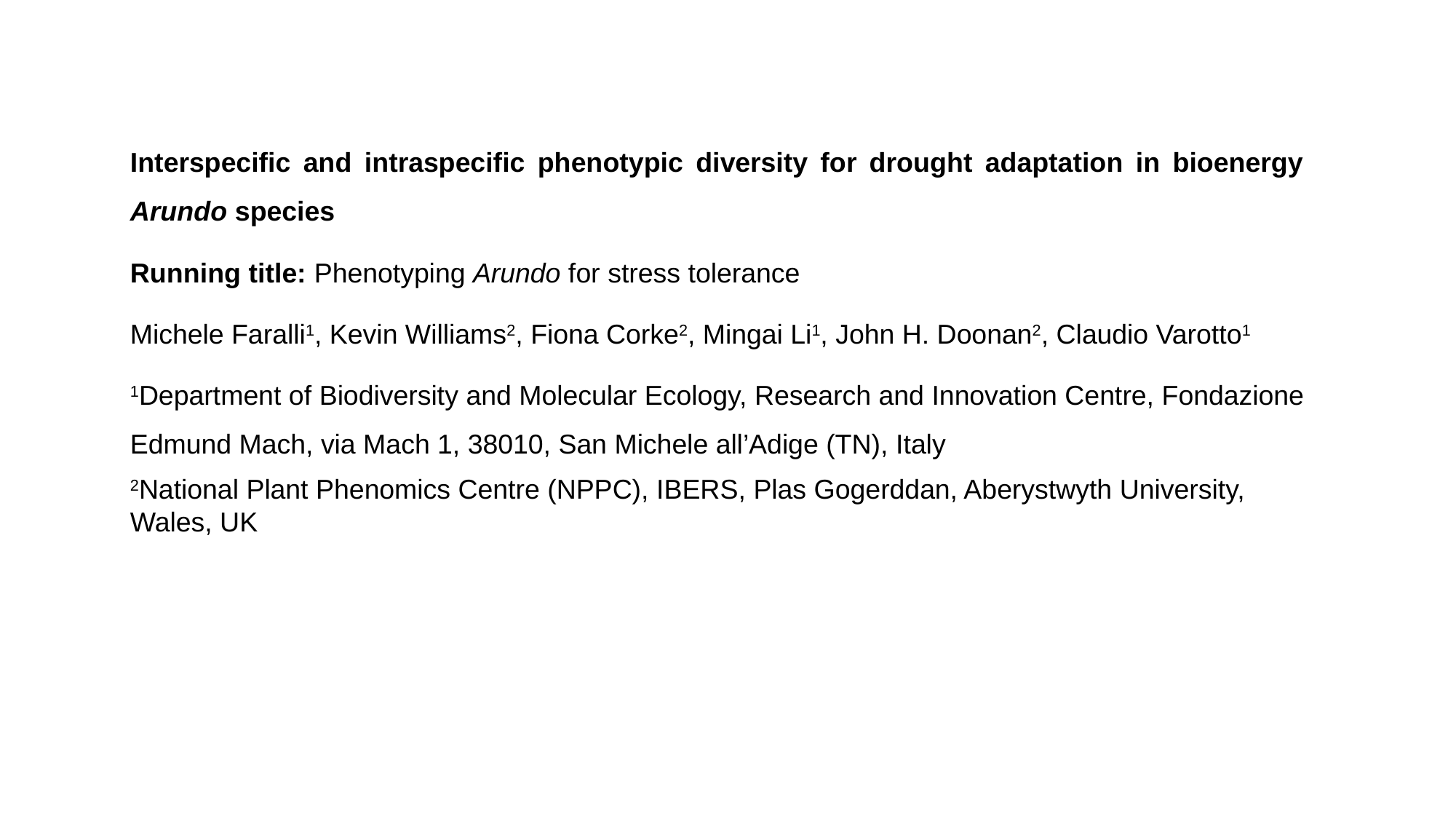

Interspecific and intraspecific phenotypic diversity for drought adaptation in bioenergy Arundo species
Running title: Phenotyping Arundo for stress tolerance
Michele Faralli1, Kevin Williams2, Fiona Corke2, Mingai Li1, John H. Doonan2, Claudio Varotto1
1Department of Biodiversity and Molecular Ecology, Research and Innovation Centre, Fondazione Edmund Mach, via Mach 1, 38010, San Michele all’Adige (TN), Italy
2National Plant Phenomics Centre (NPPC), IBERS, Plas Gogerddan, Aberystwyth University, Wales, UK

## Slide 2
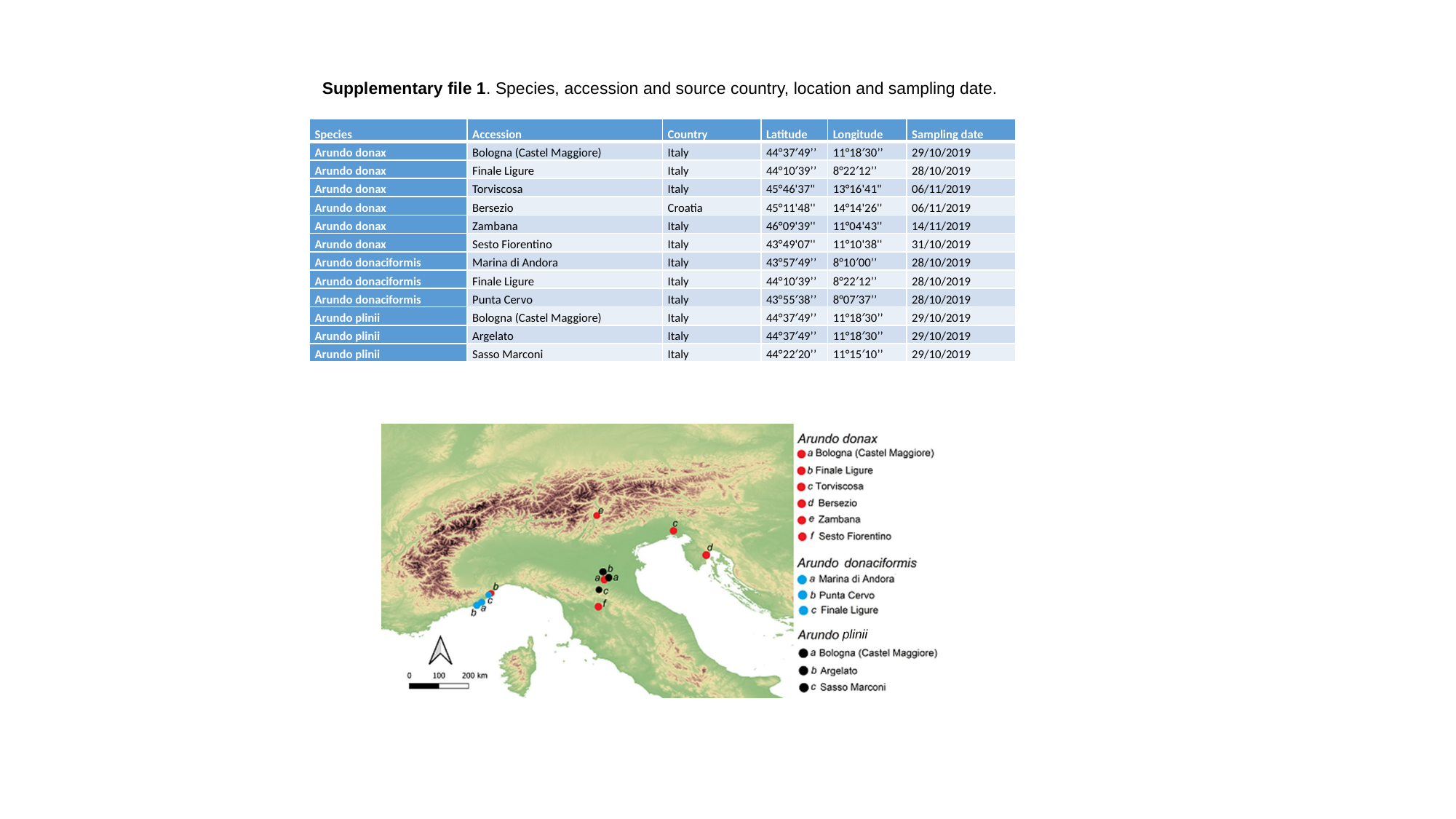

Supplementary file 1. Species, accession and source country, location and sampling date.
| Species | Accession | Country | Latitude | Longitude | Sampling date |
| --- | --- | --- | --- | --- | --- |
| Arundo donax | Bologna (Castel Maggiore) | Italy | 44°37′49’’ | 11°18′30’’ | 29/10/2019 |
| Arundo donax | Finale Ligure | Italy | 44°10′39’’ | 8°22′12’’ | 28/10/2019 |
| Arundo donax | Torviscosa | Italy | 45°46'37" | 13°16'41" | 06/11/2019 |
| Arundo donax | Bersezio | Croatia | 45°11'48'' | 14°14'26'' | 06/11/2019 |
| Arundo donax | Zambana | Italy | 46°09'39'' | 11°04'43'' | 14/11/2019 |
| Arundo donax | Sesto Fiorentino | Italy | 43°49'07'' | 11°10'38'' | 31/10/2019 |
| Arundo donaciformis | Marina di Andora | Italy | 43°57′49’’ | 8°10′00’’ | 28/10/2019 |
| Arundo donaciformis | Finale Ligure | Italy | 44°10′39’’ | 8°22′12’’ | 28/10/2019 |
| Arundo donaciformis | Punta Cervo | Italy | 43°55′38’’ | 8°07′37’’ | 28/10/2019 |
| Arundo plinii | Bologna (Castel Maggiore) | Italy | 44°37′49’’ | 11°18′30’’ | 29/10/2019 |
| Arundo plinii | Argelato | Italy | 44°37′49’’ | 11°18′30’’ | 29/10/2019 |
| Arundo plinii | Sasso Marconi | Italy | 44°22′20’’ | 11°15′10’’ | 29/10/2019 |

## Slide 3
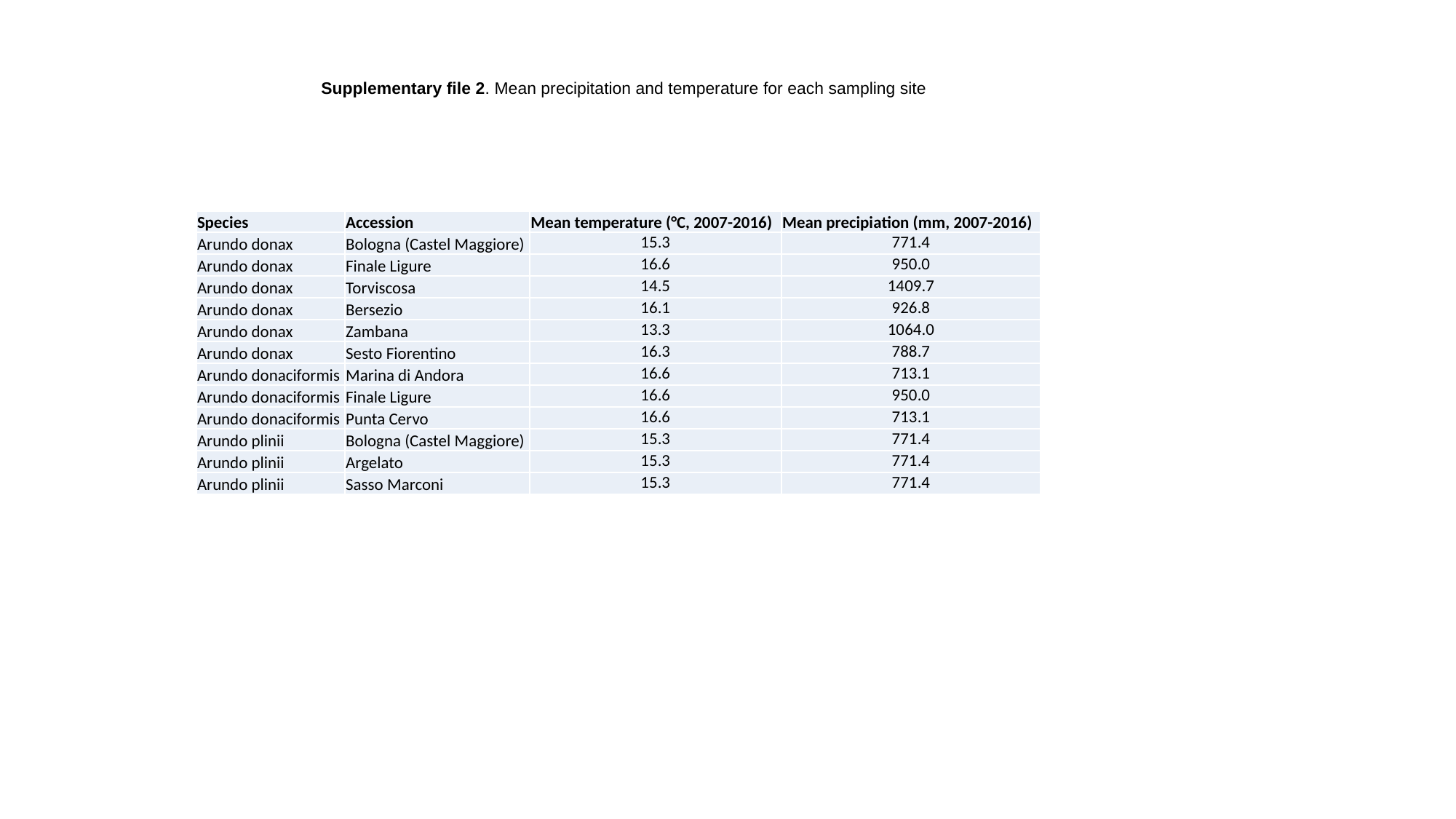

Supplementary file 2. Mean precipitation and temperature for each sampling site
| Species | Accession | Mean temperature (°C, 2007-2016) | Mean precipiation (mm, 2007-2016) |
| --- | --- | --- | --- |
| Arundo donax | Bologna (Castel Maggiore) | 15.3 | 771.4 |
| Arundo donax | Finale Ligure | 16.6 | 950.0 |
| Arundo donax | Torviscosa | 14.5 | 1409.7 |
| Arundo donax | Bersezio | 16.1 | 926.8 |
| Arundo donax | Zambana | 13.3 | 1064.0 |
| Arundo donax | Sesto Fiorentino | 16.3 | 788.7 |
| Arundo donaciformis | Marina di Andora | 16.6 | 713.1 |
| Arundo donaciformis | Finale Ligure | 16.6 | 950.0 |
| Arundo donaciformis | Punta Cervo | 16.6 | 713.1 |
| Arundo plinii | Bologna (Castel Maggiore) | 15.3 | 771.4 |
| Arundo plinii | Argelato | 15.3 | 771.4 |
| Arundo plinii | Sasso Marconi | 15.3 | 771.4 |

## Slide 4
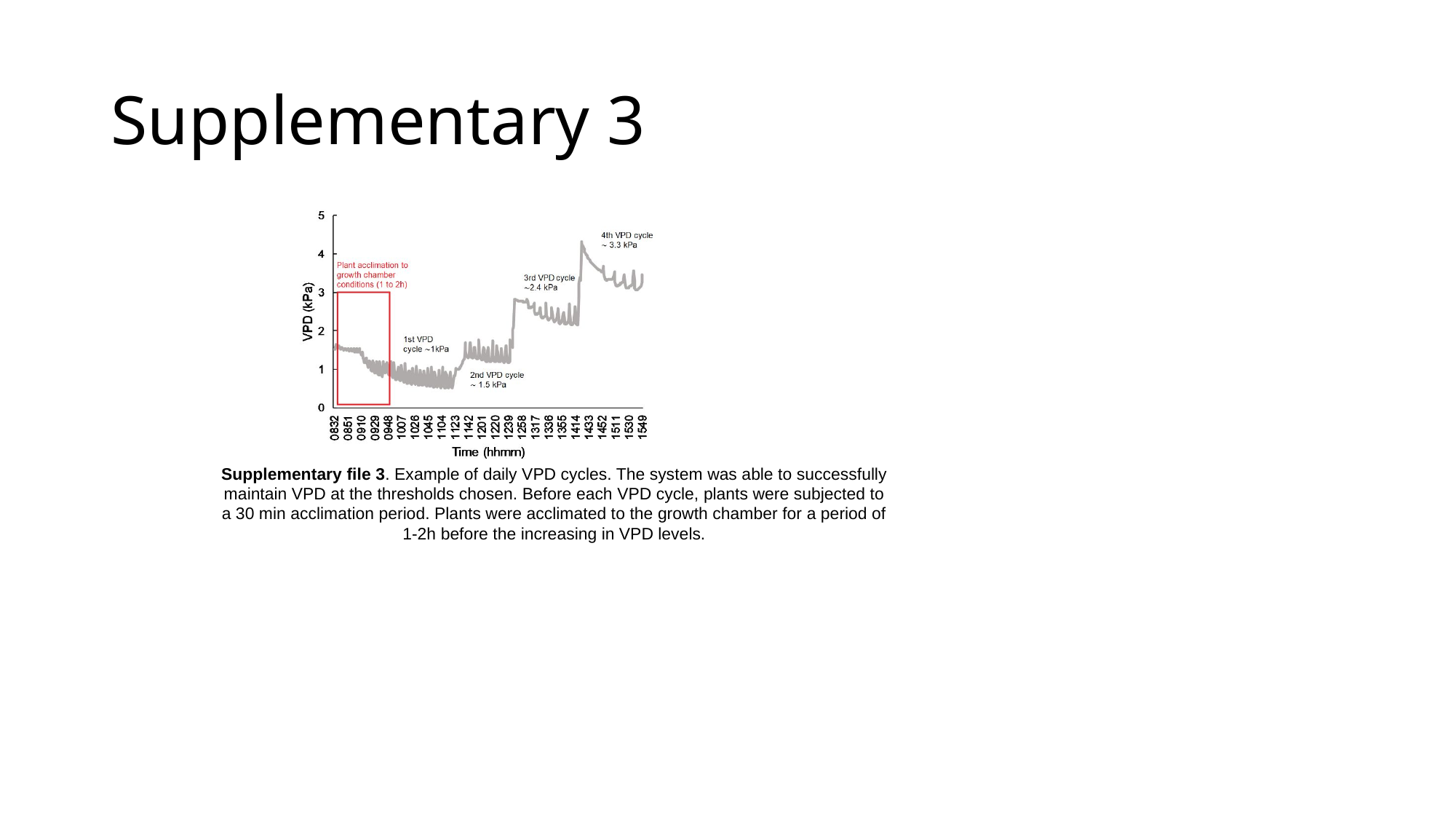

# Supplementary 3
Supplementary file 3. Example of daily VPD cycles. The system was able to successfully maintain VPD at the thresholds chosen. Before each VPD cycle, plants were subjected to a 30 min acclimation period. Plants were acclimated to the growth chamber for a period of 1-2h before the increasing in VPD levels.

## Slide 5
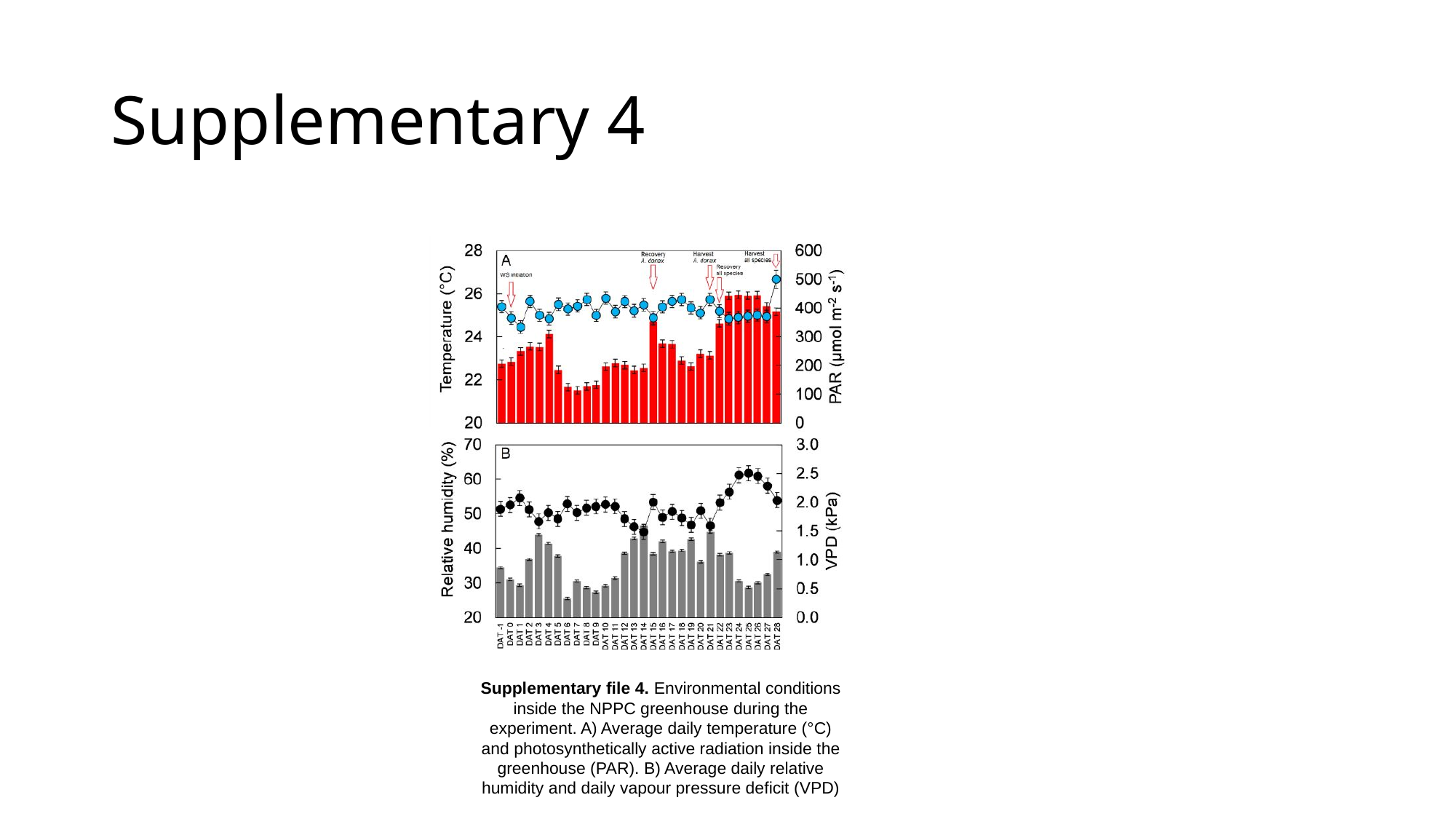

# Supplementary 4
Supplementary file 4. Environmental conditions inside the NPPC greenhouse during the experiment. A) Average daily temperature (°C) and photosynthetically active radiation inside the greenhouse (PAR). B) Average daily relative humidity and daily vapour pressure deficit (VPD)

## Slide 6
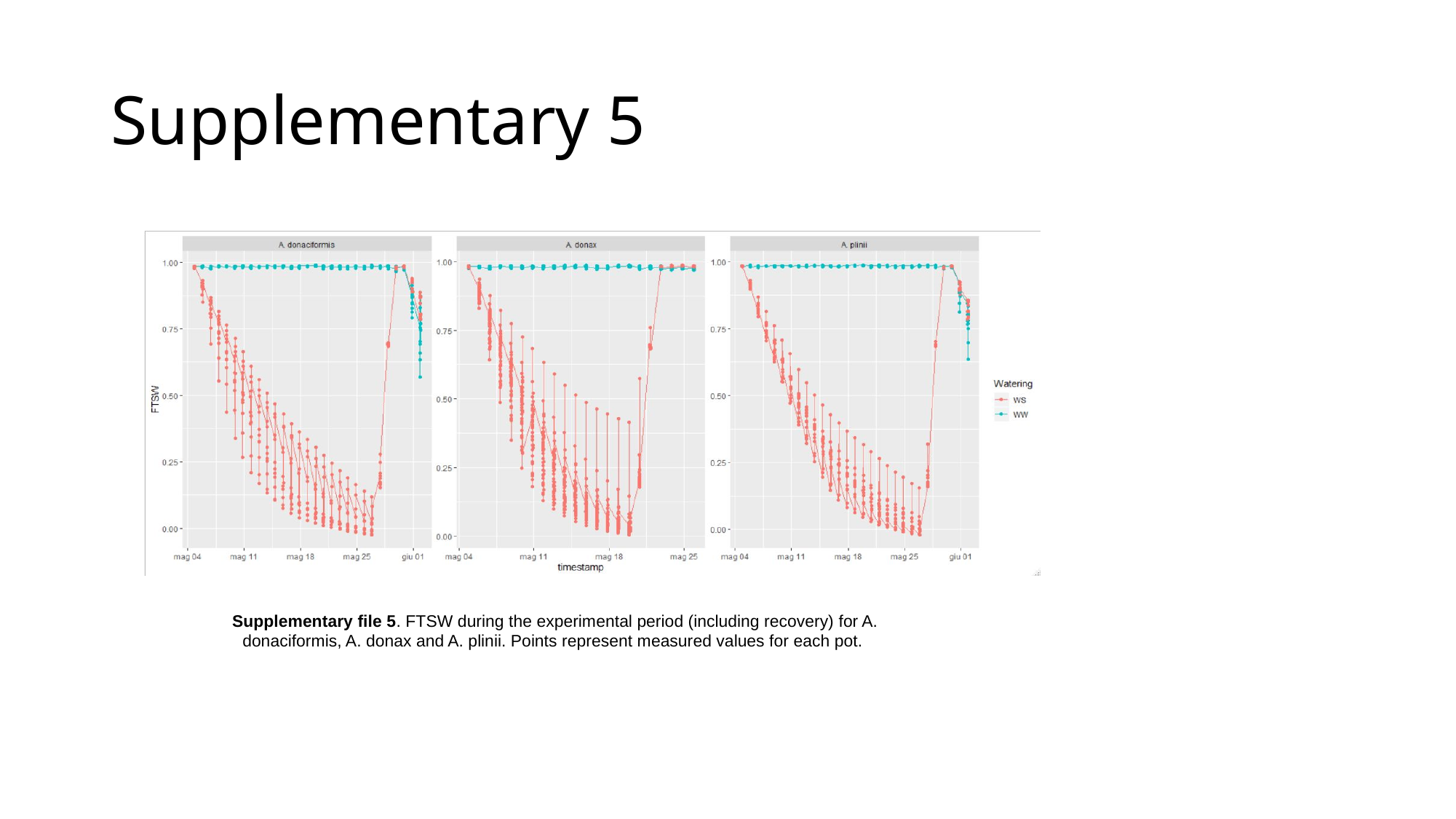

# Supplementary 5
Supplementary file 5. FTSW during the experimental period (including recovery) for A. donaciformis, A. donax and A. plinii. Points represent measured values for each pot.

## Slide 7
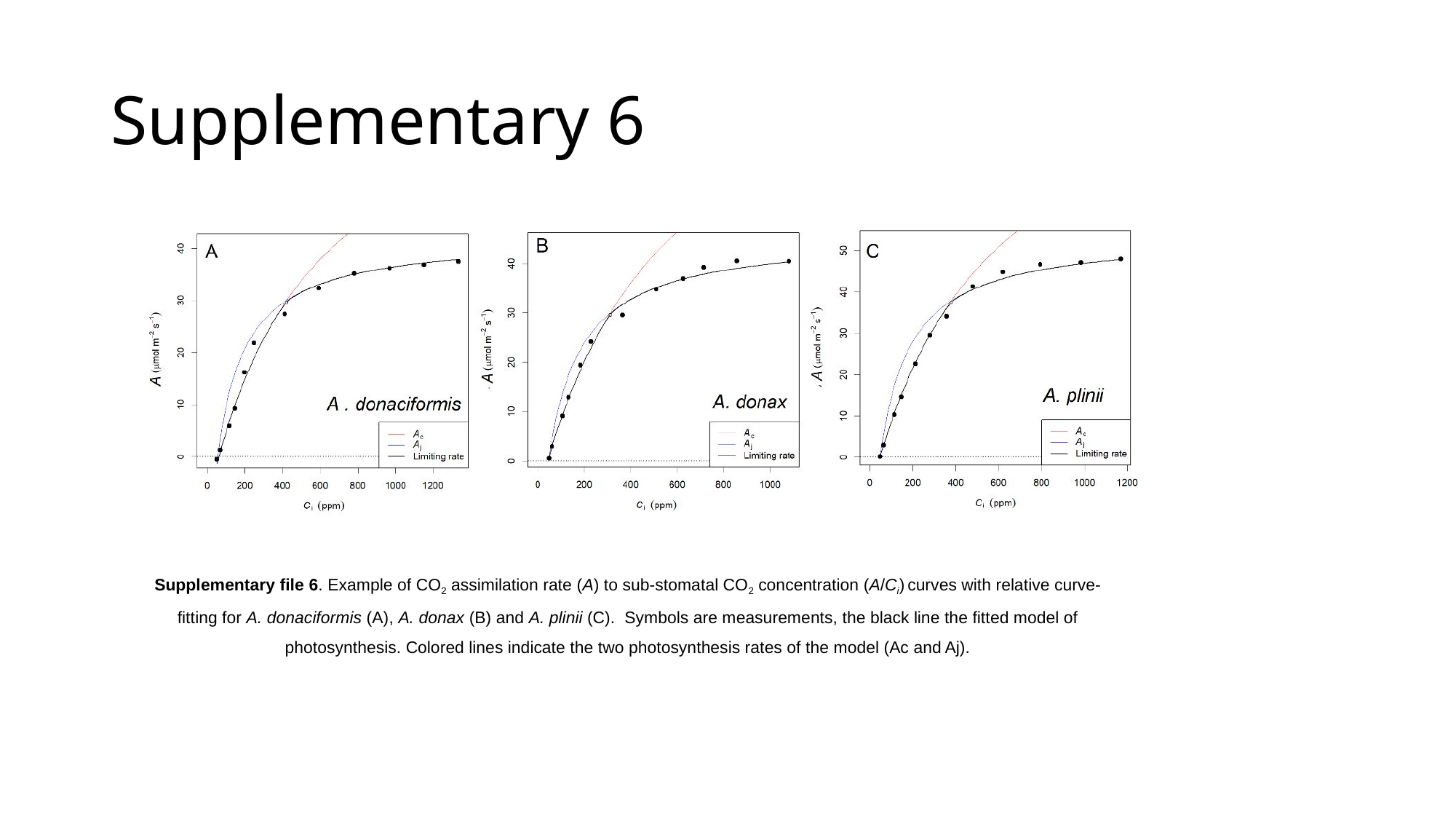

# Supplementary 6
Supplementary file 6. Example of CO2 assimilation rate (A) to sub-stomatal CO2 concentration (A/Ci) curves with relative curve-fitting for A. donaciformis (A), A. donax (B) and A. plinii (C). Symbols are measurements, the black line the fitted model of photosynthesis. Colored lines indicate the two photosynthesis rates of the model (Ac and Aj).

## Slide 8
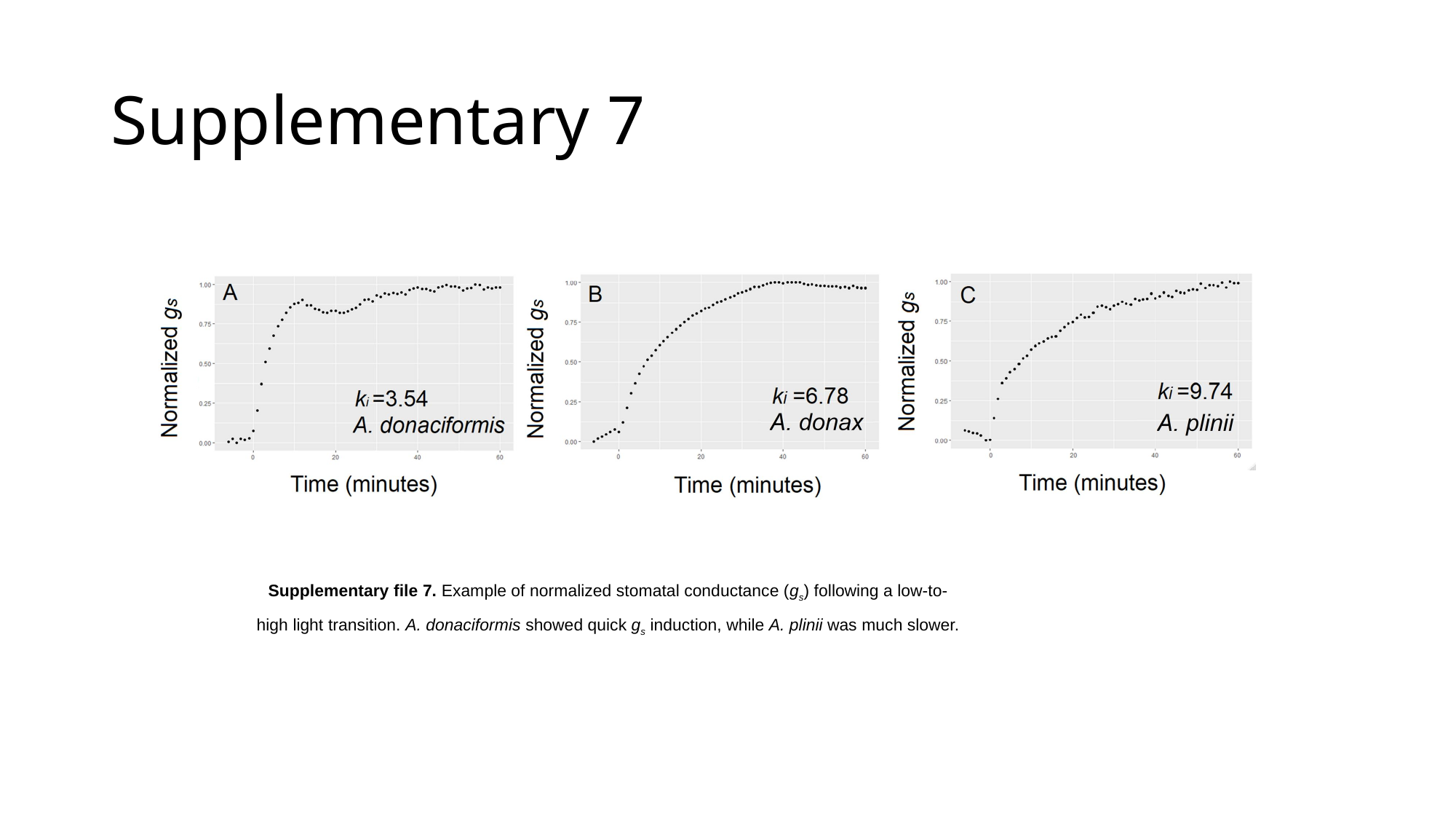

# Supplementary 7
Supplementary file 7. Example of normalized stomatal conductance (gs) following a low-to-high light transition. A. donaciformis showed quick gs induction, while A. plinii was much slower.

## Slide 9
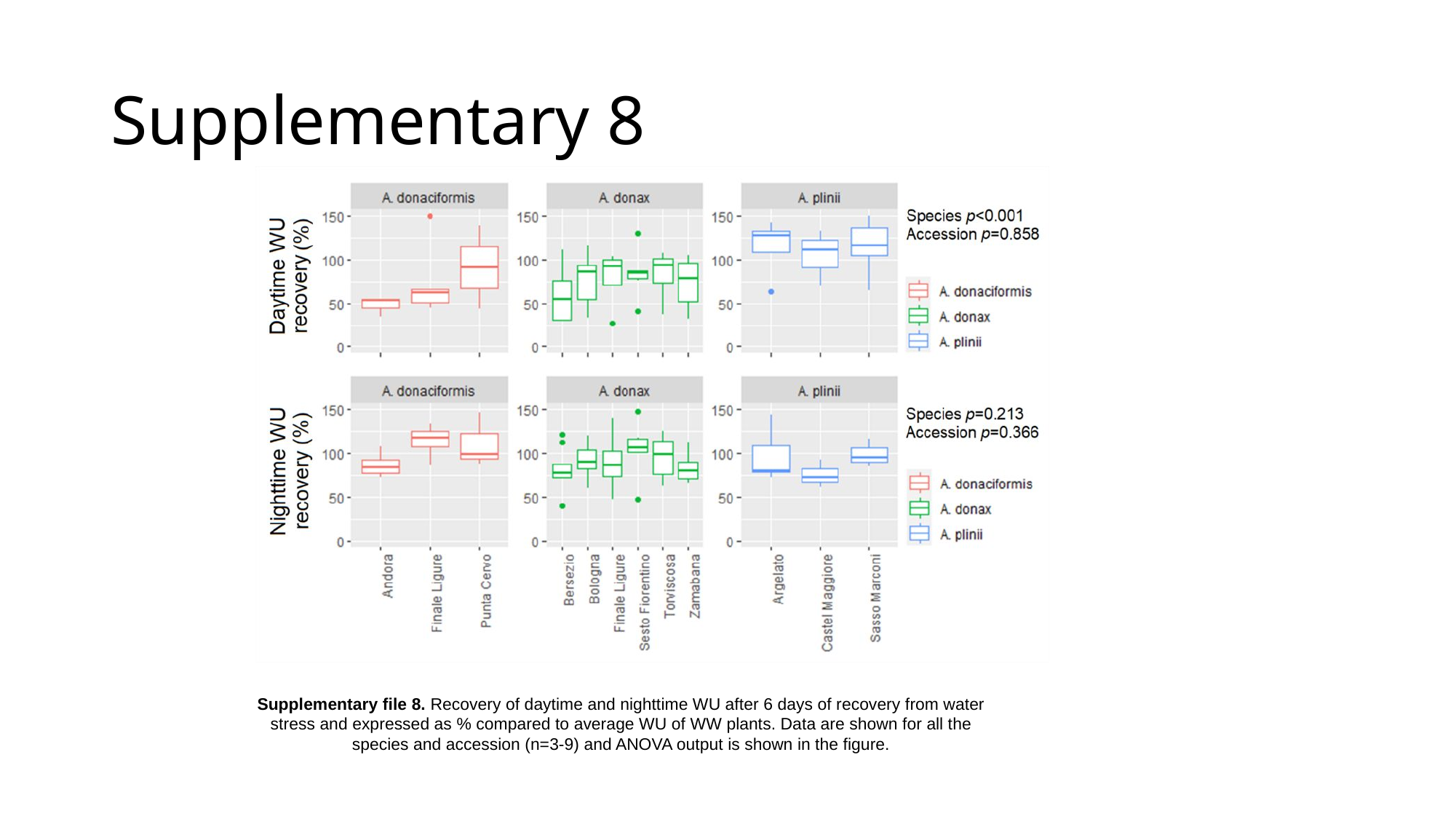

# Supplementary 8
Supplementary file 8. Recovery of daytime and nighttime WU after 6 days of recovery from water stress and expressed as % compared to average WU of WW plants. Data are shown for all the species and accession (n=3-9) and ANOVA output is shown in the figure.
